# Super-Resolved 3D Mapping of Molecular Orientation Using Vibrational Techniques

**DOI:** 10.1021/jacs.2c05306

**Published:** 2022-07-26

**Authors:** Paulina Koziol, Karolina Kosowska, Danuta Liberda, Ferenc Borondics, Tomasz P. Wrobel

**Affiliations:** † Solaris National Synchrotron Radiation Centre, Jagiellonian University, Czerwone Maki 98, 30-392 Krakow, Poland; ‡ Institute of Physics, Jagiellonian University, Lojasiewicza 11, 30-348 Krakow, Poland; § Synchrotron SOLEIL, L’Orme Des Merisiers, Saint-Aubin - BP 48, 91192 Gif-Sur-Yvette, France

## Abstract

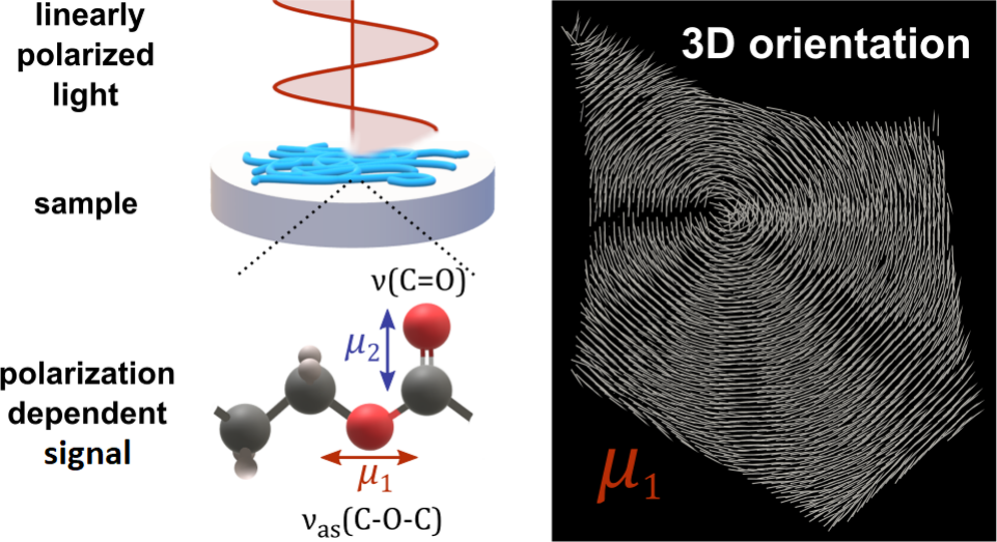

When a sample has an anisotropic structure, it is possible to obtain additional information controlling the polarization of incident light. With their straightforward instrumentation approaches, infrared (IR) and Raman spectroscopies are widely popular in this area. Single-band-based determination of molecular in-plane orientation, typically used in materials science, is here extended by the concurrent use of two vibration bands, revealing the orientational ordering in three dimension. The concurrent analysis was applied to IR spectromicroscopic data to obtain orientation angles of a model polycaprolactone spherulite sample. The applicability of this method spans from high-resolution, diffraction-limited Fourier transform infrared (FT-IR) and Raman imaging to super-resolved optical photothermal infrared (O-PTIR) imaging. Due to the nontomographic experimental approach, no image distortion is visible and nanometer scale orientation domains can be observed. Three-dimensional (3D) bond orientation maps enable in-depth characterization and consequently precise control of the sample’s physicochemical properties and functions.

## Introduction

Chemical characterization and visualization of nanoscale structures and interphases in materials like semicrystalline polymers (spherulites, nanofibers), nanocomposites, and biological systems (proteins, peptides) are critical for a deeper understanding of the correlation between the “superstructure” of materials and its physicochemical and biological properties. Reorganization at the nano-structural level even makes it possible to obtain metal-like thermal conductors from polymers, commonly known as insulators. As an example, Hu et al. presented a polyethylene film with parallelly aligned nanofibers with thermal conductivity two orders of magnitude greater than the normal polymer.^1^ Research is also ongoing into flexible energy storage based on polymer nanohydrogels,^2^ e-skin for medicine,^3^ and intelligent (biomimetic) scaffolds to induce a cellular response, e.g., stem cell differentiation,^4^ all of which originate from the molecular organization at the nano- and meso-scale. Several methods can be used to evaluate the structural and compositional properties of nanomaterials. Techniques such as X-ray diffraction probe only crystalline phases, while polarization modulation-infrared reflection–absorption spectroscopy (PM-IRRAS), calorimetry, or nuclear magnetic resonance provide very limited spatial resolution. Electron microscopy (EM) and atomic force microscopy (AFM) offer high spatial resolution at the cost of specificity. Optical spectroscopic techniques (fluorescence, Raman, infrared, or X-ray) seem to be the middle ground, providing a wealth of information while being spatially diffraction-limited,^5^ however, not offering fully three-dimensional (3D) resolved information apart for single analytes.

Fourier transform infrared spectroscopy (FT-IR) in combination with chemometrics is an intensively developed, powerful tool for the investigation of a material’s chemical structure with simultaneous microscopic visualization.^6^ One of the shortcomings of the FT-IR imaging method is the inherent low spatial resolution (∼2–10 μm), restricted by the diffraction limit close to the wavelength (λ) of light. An emerging technique that overcomes this limitation by detecting photothermal expansion caused by IR absorption using an optical probe (a visible laser) has recently been commercialized under the name of optical photothermal infrared spectroscopy (O-PTIR).^7^ This far-field microscopic technique overcomes several limitations of traditional IR microspectroscopy: sample thickness/reflective properties, optical properties of the sample substrate, or the presence of water while offering super-resolved imaging with an effective 400 nm spatial resolution.^8^

IR spectroscopy with linear polarization can provide new and useful information about the orientation of molecules.^9^ When a sample has an anisotropic structure, changing the polarization of incident light in a microscopic investigation can reveal additional information. The in-plane direction of transition dipole moments of vibrational bands can be described by the azimuthal angle (ψ), when a linear polarization-based method is applied. The IR^9^ and Raman^10^ two-polarization (2P) methods were used to examine the orientation in polymeric samples for decades, however, with significant limitations. Next, the four-polarization method (4P) has been proposed, which enabled the study of the in-plane organization of molecules in complex systems. The first applications of IR imaging with 4P on polymeric systems^11−14^ were followed by active compound visualization^15^ and recently by human tissue microarray analysis by Koziol et al. in 2020.^16,17^

Until now, experiments focused on using a single vibrational band to retrieve the in-plane orientation and internal ordering of samples. 4P is experimentally a simple (no sample tilting) framework that offers much more with proper mathematical treatment. A concurrent analysis of two vibrational bands of roughly perpendicular transition moment orientations was suggested and results in full 3D angles (in-plane and out-of-plane) retrieval.^18^ We show here, to the best of our knowledge, the first application of concurrent analysis (4P-3D) to IR imaging data and obtain orientation angles of a model spherulite polycaprolactone sample. Independently, Xu et al. followed with two examples of concurrent 3D orientation application to IR imaging.^19,20^ Moreover, we show that this method can be applied to diffraction-limited FT-IR and Raman imaging and even to super-resolved O-PTIR imaging. Due to the stationary experimental approach, no image distortion is visible and nanometer scale domains can be observed.

## Results and Discussion

**Figure 1:**
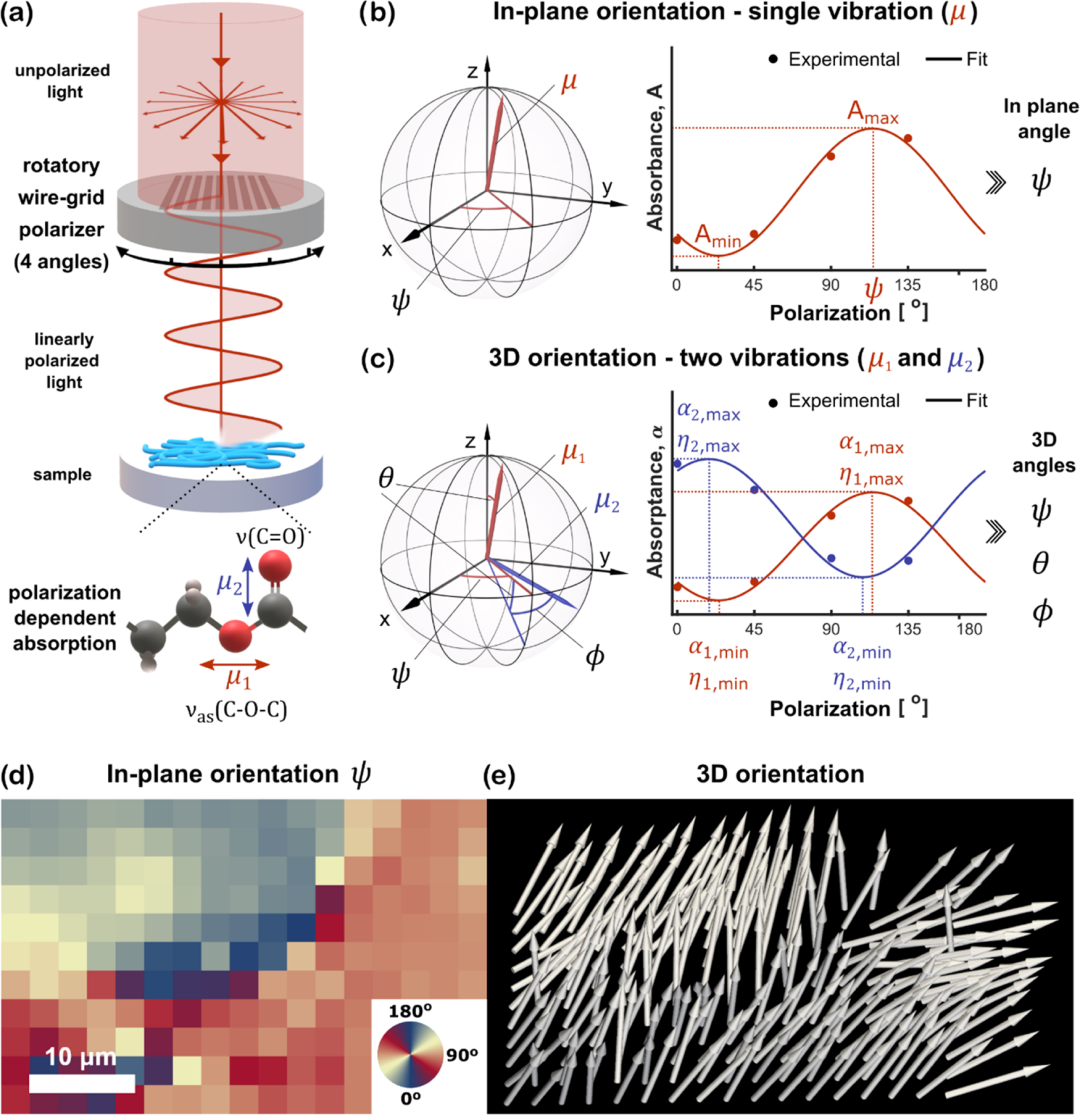
Orientation determination approach. (a) Schematic drawing of polarized FT-IR experimental setup including absorbance dependence of a molecule from the orientation of incident polarization. (b) Visualization of the 4P method used to determine in-plane orientation (2D) of transition dipole moment μ. (c) Visualization of the 4P method used to determine 3D orientations of transition dipole moments μ_1_ and μ_2_. (d, e) Examples of visualization results for 2D and 3D methods, for the same sample region, respectively.

Figure 1 presents the concept of the 4P-3D analysis (briefly described in the Materials and Methods section) leading to the retrieval of 3D orientation. Four different linear polarization angles allow probing a sinusoidal function that arises from orientation-dependent absorbance. The analysis of a single vibration leads to obtaining a 2D in-plane angle of orientation, while simultaneous analysis of two near-orthogonal vibrations retrieves 3D angles.

### PCL Spherulite Model Sample

**Figure 2:**
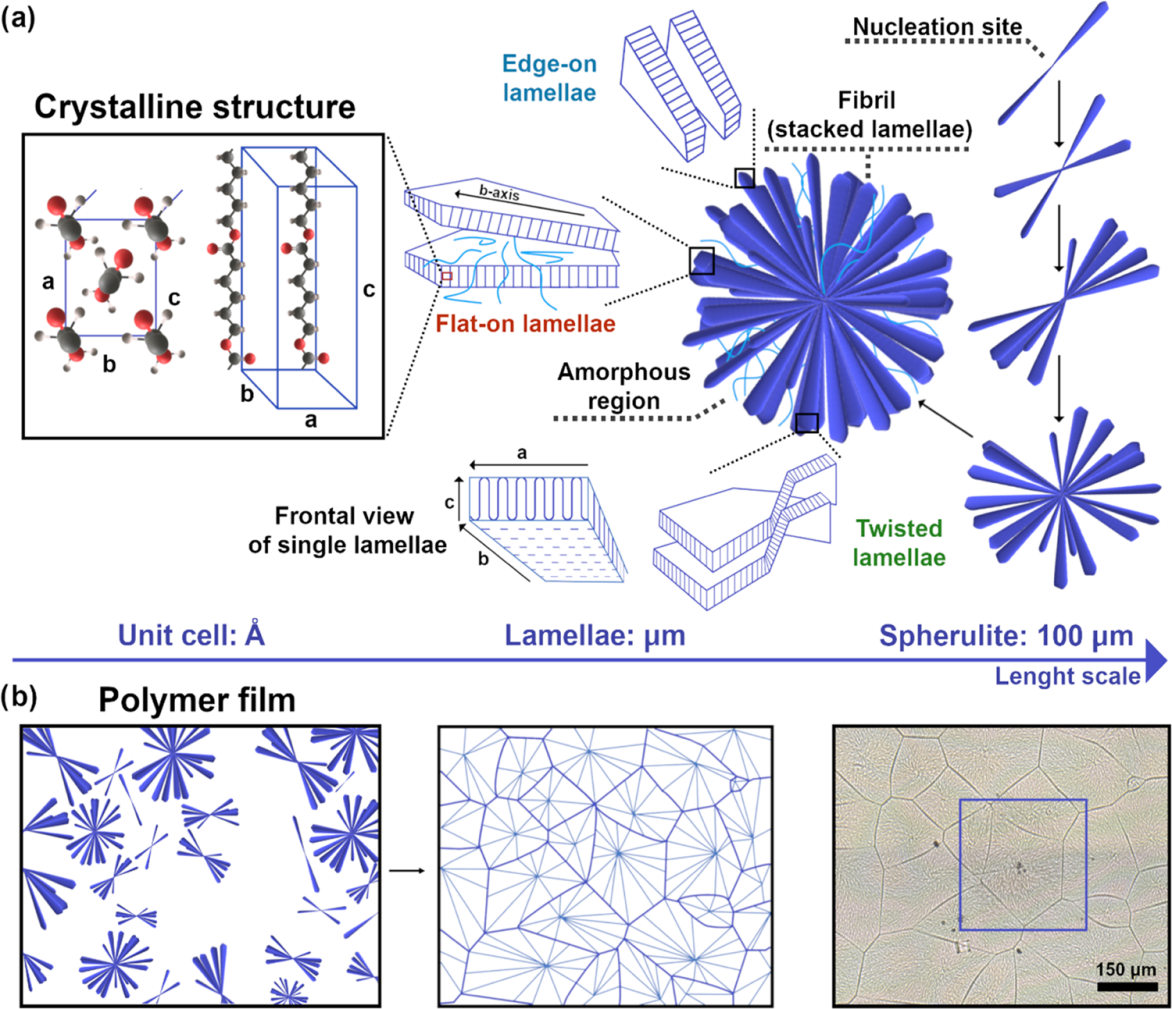
Schematic representation of the hierarchical structure of the model PCL polymer spherulite. In panel (a), the orientation of polymer chains and crystalline unit cell of PCL is indicated together with the crystal axes of the lamella made of tightly folded chains. A spherulite is composed of highly ordered lamellae, connected by amorphous regions, in different orientations: the “edge-on” orientation, “flat-on” orientation, and twisted form. Spherulite growth during polymer crystallization starts in the nucleation site. The size is controlled by the nucleation of spherulites and is in the tens of μm range with an approx. 10 μm thickness. Panel (b) shows the schematic illustration of polymer film crystallization and optical microscopic image of a PCL film with a marked spherulite selected for detailed study. With a large number of nucleation sites, spherulites interact with each other upon growth.

To present the capabilities of this approach a model polymer was selected and measured using FT-IR, O-PTIR, and Raman imaging. Poly-ε-caprolactone (PCL) is often used as a model system for polymer crystallization studies due to its well-known chemical and crystal structure. PCL has an orthorhombic crystal structure with a planar, zig-zag chain conformation.^21^ In the left panel of Figure 2a the unit cell of PCL is presented. Undercooling a solution or melt of semi-crystalline polymers often produces spherulites, each with a nucleation site at its center. However, in most polymer films, spherulites appear in the flat form with radial symmetry and grow until an impact stops the process (Figure 2b). Long fibrils which are clearly visible under an optical microscope, are made of lamellae oriented in parallel and separated by amorphous regions. The free space between fibrils can be filled with the amorphous phase or remain empty. To uniformly fill the space, new fibrils must be generated during growth. Processes like heterogeneous nucleation on existing fibrils or branching generate new, neighboring fibrils (right panel of Figure 2a). Several AFM studies showed that in the early stages of growth, spherulites appear in the form of sheaves of lamellae.^22−24^ During growth crystals splay apart and fill the space.^25^ A single lamella is made of highly oriented chains folded several times, so a single chain passes through the lamellar structure more than once. This is presented in the frontal view of a single lamella section in Figure 2a. The amorphous regions between lamellae are created of folds, chain ends, and unordered structures. Chains connecting neighboring lamellae are described as “tie chains”. Lamellae grow along crystallographic axis b and they can exhibit different types of orientations on the substrate, which can change from “flat-on” to “edge-on” as shown in the middle part of Figure 2a.

### PCL Spherulite Imaging

**Figure 3:**
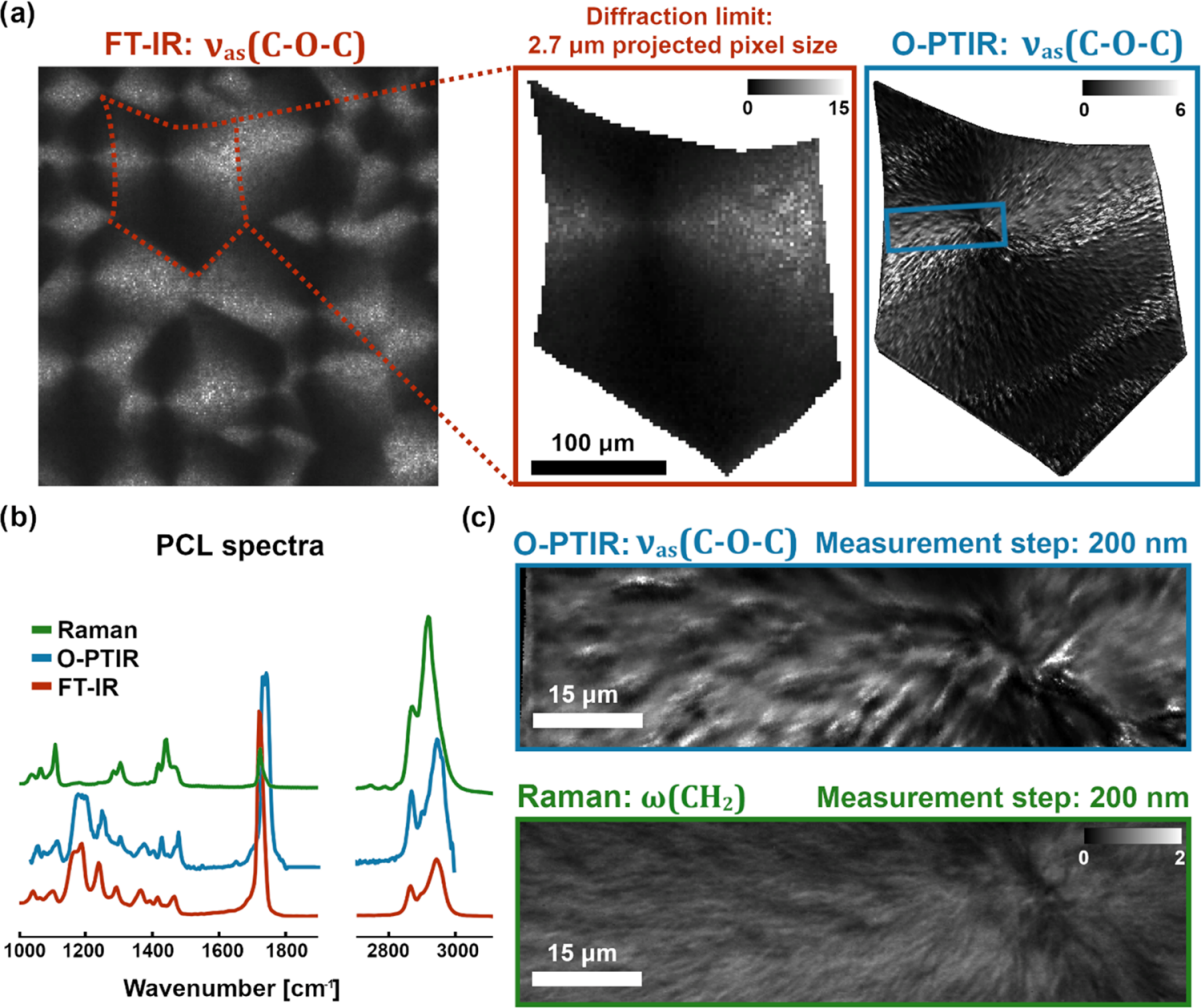
PCL spherulite imaging results. (a) Ratio of intensities corresponding to ν_as_(C–O–C) vibrations of the PCL sample, collected with two perpendicular polarizations of the incident light, using FT-IR and O-PTIR microscopes. Larger polymer film area (covering many spherulites) was acquired for FT-IR, with further attention focused on a single spherulite structure. (b) Examples of PCL spectra for FT-IR, O-PTIR, and Raman techniques (unpolarized), presenting correspondence between FT-IR and O-PTIR and the lack of some bands in the Raman spectrum. (c) Zoom of results shown above (a) for O-PTIR (blue rectangle) with corresponding results (ratio of intensities for two perpendicular polarizations) of the same sample region collected with Raman for the ω(CH_2_) band.

Data for the polycaprolactone spherulite sample described above and characterized in Figure 2 were collected using three spectroscopic techniques: FT-IR, O-PTIR, and Raman spectroscopy. Each technique possesses individual features highly influencing the measurement time, e.g., detector size or measurement step, integration time, and objective magnification. Furthermore, the measurement time is strongly increased (in comparison with experiments done with unpolarized light) by the requirement of four-polarization dataset collection. Hence, an FT-IR spectrometer with a matrix detector and lowest spatial resolution allowed the measurements of a larger area of the polymer film than other modalities, offering at the same time full spectral coverage. O-PTIR on the other hand provides significantly higher spatial detail, but to cover a region of a full spherulite, only 5 discrete frequencies were chosen for measurements. Therefore, from the whole PCL film, a single spherulite (optical image in Figure 2b) was selected for detailed analysis. Raman measurements were narrowed to a smaller spherulite region, but still covered the nucleation site and the spherulite edge. The measurement steps were kept at 200 nm in both O-PTIR and Raman and allowed us to directly compare the two datasets as shown in Figure 3c. To visualize results, including sample heterogeneity, images of intensity ratios for two perpendicular polarizations are presented in Figure 3a,c as would typically be done in the 2P approach. Specific bands were selected for each method, ν_as_(C–O–C) for FT-IR and O-PTIR, and ω(CH_2_) for Raman—both with transition moment changes approximately parallel to the main molecular axis. It is clearly visible that there is strong absorption dependence from the incident polarization direction—some regions are highlighted. Different spherulites might be recognized based on the FT-IR intensity image of the PCL film. Moreover, the nucleation site is clearly visible for all techniques. Exemplary spectra (unpolarized) from the PCL spherulite sample are presented in Figure 3b, with a good correspondence between FT-IR and O-PTIR techniques. Additionally, polarized FT-IR spectra from a single pixel are available in the supplementary data (Figure S20).

### 3D Orientation

Collected datasets were used to determine the orientation of the transition dipole moment and polarizability tensor (which is simplified to a vectorial value, due to the assumptions of the theoretical derivation),^26^ which can be used to conclude on chemical bonds and macromolecular orientations. Even though it is not the focus of this article, the results of in-plane orientation along with Hermans in-plane orientation function are presented in the supplementary materials for all three spectroscopic techniques (Figures S1–S3).

**Figure 4:**
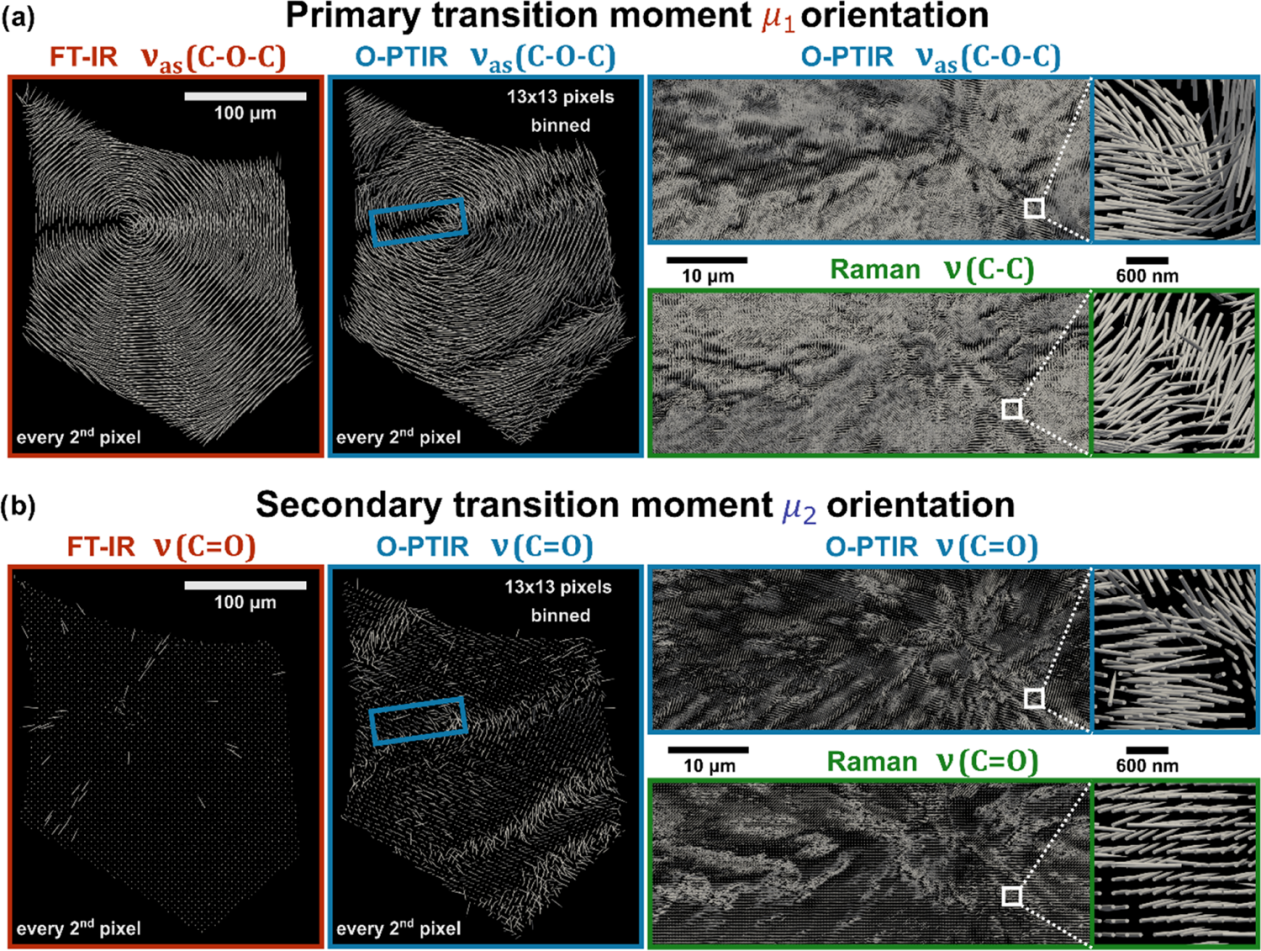
4P-3D orientation results. Visualization of the primary (a) and secondary (b) transition moment orientation calculated for FT-IR, O-PTIR, and Raman. To compare FT-IR and O-PTIR results, O-PTIR data were binned (13 × 13 pixels) to yield a similar pixel size as FT-IR. Orientation results calculated using binned intensities are presented in the middle column. To preserve visualization clarity, results for every second pixel are presented in FT-IR and O-PTIR comparison sections. Orientations for a smaller area of spherulite (blue frame covering the nucleation center) are presented for O-PTIR (without binning) and Raman in the right column, using their high detail content coming from a diffraction-limited image probed with a 532 nm laser.

As described in the theoretical section, 3D orientation calculations were done based on pairs of band intensities, corresponding to transition moments having approximately perpendicular orientations (with respect to each other). Exemplary results are presented in Figure 4 for ν_as_(C–O–C) used as a primary vector (ν(C–C) in the case of Raman) and ν(C=O) as the secondary vector. The primary vector was always chosen to be approximately parallel to the polymer chain. Unfortunately, the size of the large 3D datasets makes them hard to visualize, therefore, for clarity, in some cases, only every second point is presented. Moreover, the large pixel density along with acquired data size for O-PTIR (1500 × 1500 pixels) made it impossible to present the whole spherulite at once, therefore, the comparison to Raman data is the focus in Figure 4. Results for other pairs of spectroscopic bands and larger sample regions in O-PTIR are visualized in Figures S4–S19 in the Supporting Material.

Considering the crystalline structure of lamellae in the PCL spherulite presented in Figure 2, conclusions could be done regarding theoretical transition moment orientations. With fibrils being organized radially from the nucleation center, the direction of the main molecular axis (polymer chain) is expected to be perpendicular to the fibril axis. This prediction is confirmed by 3D orientation results for ν_as_(C–O–C) and the best visualization is provided by FT-IR and binned O-PTIR shown in the left part of Figure 4a. The purpose of O-PTIR data binning yielding a similar spatial detail (pixel size) as FT-IR was to validate and compare results provided by the two techniques. Numerical evaluation is provided in Figure S8 in the form of Euclidean distance. Orientation vectors used for data presentation form concentric circles with the center located exactly within the nucleation site area, which confirms that the polymer chain is perpendicular with respect to the fibril’s axis. The two techniques significantly differ in the optical setup and artifacts coming from scattering and varying sample thickness are also not the same. Still, the obtained results are consistent with each other, which is confirmed by the Euclidean distance results. However, the exact orientation of primary transition moment depends on lamellae alignment, which can vary from flat-on to twisted to edge-on (Figure 2a). Based on FT-IR and binned O-PTIR orientation of ν_as_(C–O–C) one may conclude that the whole spherulite consists of fibrils formed with edge-on lamellae (flat-on lamellae should exhibit an orientation perpendicular to the figure plane). Nonetheless, in this case, the pixel size (∼2.7 μm) is close to the size of lamellae, therefore, the averaging of signal coming from this region causes a significant loss of details observed. O-PTIR and Raman with much higher spatial resolution come to aid, with results for a region of the spherulite including the nucleation site (blue rectangle) presented in the right part of Figure 4a. As expected, the comparison of ν_as_(C–O–C) for O-PTIR and ν(C–C) for Raman shows great similarities. The general structure observed in FT-IR is also preserved in O-PTIR, but there is a striking increase in detail. Heterogeneity of the sample is clearly visible with wavy structures probably corresponding to fibril shapes. At such magnification, it cannot be stated if the spherulite is built exclusively from edge-on lamellae, because both parallel and out of the figure plane vectors are observed in different sample regions.

Interesting results are also observed for the secondary transition moment (Figure 4b). The ν(C=O) vibration should be approximately perpendicular to the molecular chain, but it is hard to predict their exact theoretical orientation even if lamellae alignment (edge-on or flat-on) would be determined. Therefore, it is interesting that for FT-IR and binned O-PTIR (left sector of Figure 4b) vectors are consistently oriented perpendicularly to the figure plane. There is a slight tilt in O-PTIR results in comparison with FT-IR, but the vectors’ general trend is the same, and differences could be again caused by the dissimilarities in optical setups. Raman and O-PTIR once more show analogous results, preserving a perpendicular trend with respect to the primary vector. Although, vectors are no longer consistently perpendicular to the figure plane and heterogenic wavy shapes can be again observed. A detailed relation between primary and secondary vectors might be observed in zoomed-in areas for O-PTIR and Raman.

Combining orientation information of primary and secondary vectors, one may conclude that the investigated spherulite is primarily built out of edge-on lamellae. First, for all observed techniques, primary vectors are mostly aligned or slightly out of the figure plane, which theoretically should be expected for edge-on units. This hypothesis is supported by the perpendicular character (to the figure plane) of secondary vectors. Obviously, there are regions exhibiting those properties to a different extent, but this is expected since the crystallization process is very subtle, and its conditions have a major influence on lamellae alignment.

Valid information about cylindrical symmetry and molecular ordering is provided by the ⟨*P*_2_⟩ parameter, with results shown in supplementary materials (Figures S4–S7 and S9–S19). As mentioned in the 3D part of the Theoretical Approach section (Materials and Methods), ⟨*P*_2_⟩ is used as a metric describing the breadth of orientation distribution function (ODF), e.g., ⟨*P*_2_⟩ = 1 for infinitely narrow distribution parallel to the symmetry axis, whereas ⟨*P*_2_⟩ = 0 in the case of an isotropic distribution. Negative values reaching ⟨*P*_2_⟩ = −1/3 characterize distributions perpendicular with respect to the symmetry axis. Considering that primary transition moments used in this study were always chosen to be approximately parallel to the main molecular axis, the analysis of ⟨*P*_2_⟩ results may lead to conclusions in which bands are the best indicator of the main molecular axis. Based on FT-IR and O-PTIR results (Figures S4–S7 and S9–S14), the highest ⟨*P*_2_⟩ values are reached by ν(CC–O) vibrations. Additionally, close to zero results (⟨*P*_2_⟩ = 0) are observed (FT-IR data) around the nucleation center, which confirms its amorphic character. The smallest breadth of ODF in the case of Raman is observed for the ν(C–C) vibration associated with 1037 cm^–1^.

**Figure 5:**
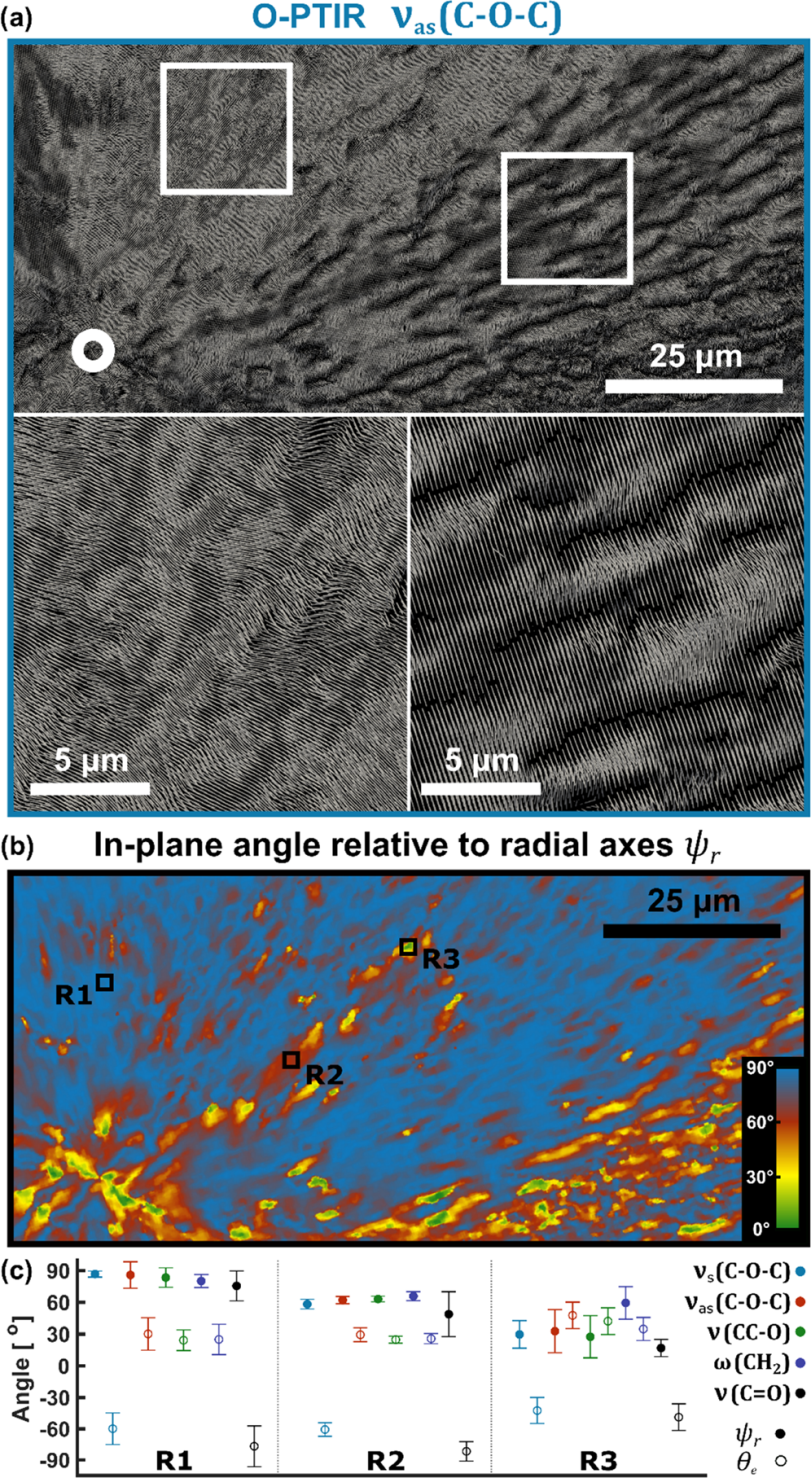
4P-3D orientation analysis based on O-PTIR. (a) Orientation of the ν_as_(C–O–C) dipole moment based on the O-PTIR dataset. Zoomed areas are pointing to two clearly distinguishable sample regions, which may be related to areas of different spherulite growth phases. Nucleation site is marked with a white circle. (b) In-plane orientation relative to the radial axes ψ_r_ for ν_as_(C–O–C). (c) Mean values of ψ_r_ and θ_e_ for three regions (R1, R2, and R3) for different dipole moments along with standard deviations within that region.

Molecular orientation (main polymer axis) and lamellae alignment based on O-PTIR were so far only discussed on a limited region of the sample corresponding to the Raman measurement area to allow for a direct comparison. A larger area presenting the orientation of ν_as_(C–O–C) is shown in Figure 5a with more structural insight. Once more, the presented region includes the nucleation site marked with a white circle, as it is the most characteristic spot in spherulites. Having a look at spherulite’s larger area one can notice that there are two significantly different domains with zoom-ins marked with a white box. Interestingly, after overlaying the orientation image presented here (right zoom) with the optical microscopy image, fibrils are overlapping with structures formed by vectors (results not shown). However, such structures are not observed in the left zoom where vectors are more parallel to each other. This is regardless of the fact that fibrils observed in the optical image have the same structure, with no visible differences. This may lead to a conclusion that there are significant differences within fibril structures on the molecular level, probably associated with growth phases. As explained earlier (Figure 2a), at the early stages of spherulite formation there is a single fiber, which later becomes surrounded by other radially oriented fibers and branches until full spherulite is formed. For a polymer film with a thickness larger than 500 nm, the edge-on orientation of lamellae is expected. Single lamella is built of tightly packed and folded polymer chains. In the commonly accepted model, the chains are aligned normal to the plane of the lamella, so the orientation of chains determines the orientation of the lamella. In the spherulites, lamellas are stacked together, creating thicker structures, the fibrils (Figure 2). The unambiguous determination of lamellae orientation requires further development of the 4P-3D analysis method, however, obtained results give us partial information. Normal alignment of the secondary vectors to the image plane indicates more edge-on than flat-on orientation of lamellae. However, the tilt angle of lamellae requires additional modeling methods.^27^ Yet, the observed dissimilarities can arise directly from growth stages.

To have a numerical quantity allowing to visualize and compare some regions of spherulite, a relative in-plane orientation ψ_r_ was defined as the difference between the ψ angle and the direction of fibers. ψ_r_ describes an in-plane angle relative to the radial axes formed by spherulite’s fibrils. Exemplary results for the ν_as_(C–O–C) vibration (O-PTIR) are shown in Figure 5b. The presented region directly corresponds to the orientation in Figure 5a. As expected, the majority of results exhibit values of around 90° (R1 region), apart from the nucleation center, which is amorphous. Nevertheless, there are some regions with different orientations—R2 and R3. Mean values of ψ_r_ for all three regions are presented in Figure 5c, including the orientation of other vibrations. Additionally, the values for the  angle (elevation) are also presented. A combination of these results allows us to analyze relative orientations of specific transition dipole moments. Orientation of ν_as_(C–O–C) is parallel to the PCL polymer main chain axis, whereas ν_s_(C–O–C) should be perpendicular.^28^ Here, both vectors have similar values of ψ_r_, but θ_e_ differs by around 90° for all three regions, which confirms the perpendicular character of those two. Very similar results are observed for the ν_as_(C–O–C) and ν(C=O) pair. The ν(CC–O) vibration is slightly more complex, coming from C–O and C–C stretching in the crystalline phase,^28^ but the obtained results show that the transition dipole moment directions are parallel to the main chain axis, similar to ν_as_(C–O–C). The same orientation is also observed for ω(CH_2_). Thus, those three may be considered as vibrations describing the main molecular axis orientation.

## Conclusions

Here, we successfully demonstrated for the first time the four-polarization methods coupled to concurrent vibration analysis (4P-3D) on a spherulite polymer sample. We showed that this framework gives consistent results across the spectroscopic modalities, allowing diffraction-limited and even super-resolved imaging. Our research opens the way to applications for spatially resolved orientation studies of a wide range of thin film samples, in a nondestructive, label-free manner. We expect the 4P-3D method to impact both material and biological sciences as previously unattainable information can be extracted without a complicated experimental setup.

## Materials and Methods

### Experimental Approach

#### Spherulite Sample Preparation

Polycaprolactone (PCL) of *M*_n_ = 80 000 g/mol was purchased from Sigma Aldrich Sp. z o.o (Poznan, Poland). Chloroform for analysis (99.8%), amylene stabilized was obtained from Idalia Sp.J (Radom, Poland). PCL has a melting point (*T*_m_) of 60 °C and glass transition temperature (*T*_g_) of around −60 °C. The PCL film was prepared from solution (1.2 g of granular polymer in 80 mL of chloroform). The system was left on a magnetic stirrer for 24 h at room temperature to allow complete dissolution. The PCL film with a thickness of around 10 μm was prepared by casting solution onto a calcium fluoride (CaF_2_) slide. It was placed in a fume hood to evaporate the solvent overnight. PCL was melted at 75 °C to erase the structure on a hot stage and crystallized under isothermal conditions at 35 °C. After 5 h of spherulite growth, the sample was rapidly cooled down to room temperature.

#### FT-IR Imaging

FT-IR measurements were performed using a Bruker Vertex70v spectrometer coupled with a Hyperion 3000 microscope equipped with a 64 × 64 element FPA MCT array detector. A 15× Cassegrain objective (NA = 0.4) was used giving a 2.7 μm x 2.7 μm projected pixel size. To perform measurements with linearly polarized light, two wire grid polarizers were inserted before the objective and at the bottom of the condenser. Simultaneous rotation of both polarizers aligned in parallel allowed achieving desired polarizations—four datasets were collected, with relative polarizations corresponding to 0, 45, 90, and 135°. To provide pixel-to-pixel spatial alignment of all datasets, a motorized sample stage was programmed to repeat the same positions (for each polarization). Data acquisition was done in the 3850–900 cm^–1^ spectral range, with 8 cm^–1^ spectral resolution and zero filling factor of 1. Sample and background spectra were co-averaged 4 and 64 times, respectively. Background datasets were collected individually for each polarization, in a blank region of the CaF_2_ slide.

#### O-PTIR Imaging

O-PTIR imaging was done using a mIRage O-PTIR microscope. A quantum cascade laser (QCL) was used as a pump beam with a pulse rate of 100 kHz in a 2.4% duty cycle. Images were collected at five frequencies: 1736, 1368, 1296, 1246, and 1170 cm^–1^ with corresponding QCL laser powers: 4, 24, 24, 18, and 18%, respectively. A 532 nm laser with 0.5% power (about 7.5 mW power on the sample) was used as a probe beam. Measurements were performed in the reflection mode using an avalanche photodiode detector (APD) and 200 nm steps. The background was measured once, on a low-e slide (Kevley Technologies). Since QCL laser is intrinsically linearly polarized, to obtain different relative polarizations the sample was rotated roughly by 45° increments to obtain a four-polarization dataset at relative 0, 45, 90, and 135° angles. The measurement region was adjusted each time the sample was rotated, to cover the whole spherulite of interest.

#### Raman Imaging

Raman measurements were performed using a WITec confocal CRM α 300 Raman microscope with a 50× objective. The spectrometer was equipped with an air-cooled solid-state laser operating at 532 nm (with linear polarization) and a charge-coupled device (CCD) detector. The laser power measured before the objective was approximately 50 mW. The spectra were recorded with 4 cm^–1^ spectral resolution and 0.1 s integration time. An achromatic half-wave plate was placed above the objective to control the natural laser polarization direction. Four polarizations of interest (−30, 0, 60, and 120°) were achieved by the rotation of the wave plate. A rectangular fragment of the spherulite from the border of the neighboring spherulite to the nucleation site was measured with 200 nm steps (size: 150 × 450 steps).

### Preprocessing

#### FT-IR Data

Spectral and spatial data quality was increased using minimum noise fraction (MNF) denoising, with 15 bands used for reconstructions.^29,30^ Furthermore, a local linear baseline was applied to the spectral ranges corresponding to vibrations of crucial chemical bonds, which allowed the mitigation of scattering artifacts. Single intensities (within corrected ranges) were used for transition dipole moment orientation calculations, with the attention focused on 1720, 1365, 1292, 1238, and 1165 cm^–1^, coinciding with the O-PTIR data. Mentioned band identification for both IR methods is presented in Tables S1 and S2.

#### O-PTIR Data

Since for O-PTIR imaging different linear polarization angle data were collected by sample rotation (not by polarization control), an additional preprocessing step was required to spatially align four datasets. The final sample orientation was defined by the 90° polarization dataset (closest to FT-IR results orientation), whereas 0, 45, and 135°, datasets were warped by global rotation and translation of each image. Calculation of rotation angles (85.7, 136.9, 42.5°) used for image transformation was done using the line marked on the sample before measurements. Following, images were translated referring to characteristic sample structures—spherulite edges. During data manipulation, we noticed that intensities for the last polarization dataset (135°) were significantly higher and therefore not comparable to other polarizations. To overcome this artifact, all images collected with the O-PTIR technique were divided by their median value. Finally, aiming to prepare data for further absorptance calculations in the 4P-3D orientation method, intensities for each spectral variable (cumulative data for all four polarizations) were rescaled to span within the intensity range corresponding to absorption values exhibited by FT-IR data. Furthermore, additional datasets were generated by binning 13 × 13 pixels (giving 2.6 μm pixel), with the aim to later compare O-PTIR and FT-IR data (2.7 μm pixel).

#### Raman Data

The data treatment started with cosmic spike removal and Raman shift calibration to Si bands. This was followed by denoising by principal component analysis (PCA), with 15 principal components used for reconstructions, which significantly increased spectral and spatial data quality. Global baseline correction was done using the rubberband method with 13 spectral ranges used for linear corrections. One of the polarization datasets exhibited slightly higher global intensities (distorting orientation calculations), therefore, each spectrum was normalized to its sum. Similar to O-PTIR, Raman spectra also required intensity rescaling for further calculations. However, due to the absence of some bands present in the FT-IR spectra, data were scaled so that intensities for the most intensive band in Raman spectra (2917 cm^–1^) are similar to the absorbance of the most intensive bands in FT-IR data (1720 cm^–1^). Identification of Raman bands used in further analysis is available in Table S3.

### Theoretical Approach

Anisotropic materials, such as fibers or polymers with long molecular chains exhibit high absorption dependence on the polarization direction of the incoming light, both in IR spectroscopy and other spectroscopic techniques. This effect, called linear dichroism, is extensively applied in molecular orientation studies. In polarized infrared spectroscopy (FT-IR and O-PTIR), absorption by a specific molecular vibration is the highest when the transition dipole moment  vector is aligned with the polarization of light.^31^ A schematic example of an FT-IR instrumentation setup is shown in Figure 1a. Similarly, in polarized Raman spectroscopy scattering reaches a maximum for the best alignment of polarization with the highest polarizability tensor change , resulting in maximum band intensity observed in the Raman spectrum.^32^ Hence, the analysis of spectra collected for different incident light polarizations can provide information about the chemical bond and molecular orientation, with the dichroic ratio (*D*) being one of the most widely used parameters. *D* is defined as the ratio of absorbance (or intensity) for parallel and perpendicularly polarized light. However, in the case of Raman, we define *D* as a ratio of scattering intensities for perpendicular polarizations of the incident green laser used for Raman excitation, as opposed to the depolarization ratio, which compares the scattered photons. Thus, using only two polarizations introduces a specific measurement condition—parallel and perpendicular orientation of transition moment with respect to polarization. In other cases, *D* would exhibit misleading values, like in a situation with 45° angle, where both polarizations would be affecting the sample, in the same way, giving *D* = 1 and incorrectly suggesting isotropic properties. Such measurement geometry makes this method appropriate only for systems with molecules aligned alongside each other and well-known chemical bond orientation, excluding systems with molecules changing directions throughout the sample.

#### Four-Polarization Method for 2D Orientation

The disadvantages of the two-polarization method have been aided by the development of the four-polarization approach, which allows molecular bond orientation determination, independently from the choice of incident light polarization. Absorbance dependence from linear polarization may be described by a simple formula, as follows^12,13,33^
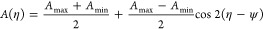
where *A*_min_ and *A*_max_ are minimum and maximum absorbance, η is the incident polarization angle and ψ is the polarization angle when *A*(η) = *A*_max_. Collecting data with four different linear polarizations (for each pixel) gives the possibility to experimentally find the shape of *A*(η), as shown in Figure 1b. The mathematical approach applied here is based on a nonlinear fit of a cosine function to extract *A*_min_, *A*_max_, and ψ. Considering dipole moment μ being oriented in 3D space, ψ gives limited information about the in-plane orientation of this vector. Exemplary result visualization (Figure 1d) clearly shows the disadvantage of the 2D method, where extracted information fully omits the out-of-plane component of the orientation. Based on *A*_min_ and *A*_max_, one can also compute Hermans (in-plane) orientation function defined as^12,13,33^
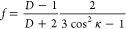
where *D* = *A*_max_/*A*_min_ and κ describe the angle between the main molecular axis and transition moment (used values are available in Tables S1–S3). Hermans function is equal to the second-order coefficient of a series of Legendre polynomials approximating the orientation distribution function (ODF) of a molecule with cylindrical symmetry.^12,18^ Therefore, it can be used to describe the level of molecular orientation of the sample. This approach is equally suitable for the analysis of FT-IR and Raman measurements, with absorbance and scattering intensity being the corresponding results.

#### Four-Polarization in 3D Orientation

Out-of-plane orientation issues, mentioned in the above section, were recently approached by Lee, who proposed a mathematical method allowing determination of 3D angles and order parameter for molecular orientation imaging with FT-IR.^18^ Derivation is established on the grounds of classical electromagnetic wave theories and concurrently uses two nonparallel transition dipole moments: the primary (μ_1_) associated with the main molecular axis and a secondary (μ_2_) representing nonparallel vibration. Based on this, the absorptance (α = 1 *– T*) dependence from the polarization direction η for vectors μ_1_ and μ_2_ is described as^18^
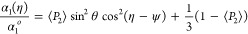

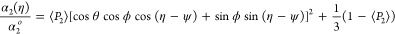
where ψ, θ, and ϕ are angles describing the 3D orientation of μ_1_ and μ_2_ vectors (shown in Figure 1c), ⟨*P*_2_⟩ is the order parameter, whereas α° contains only nonorientational quantities. Similar to the 2D approach, nonlinear functions were fitted to four-polarization datasets. This allowed the reconstruction of the experimental shape of absorptance dependence on incident polarization. As presented in Figure 1c, the following parameters pairs were extracted from the obtained experimental functions: α_1,max_, η_1,max_, α_1,min_, η_1,min_, α_2,max_, η_2,max_, and α_2,min_ and η_2,min_. Formulas needed for ψ, θ, and ϕ calculations are presented in supplementary materials. However, one needs to be aware of the fact, that this approach gives two undistinguishable orientation results, mirror symmetrical with respect to the *xy* plane as described in supplementary materials. The order parameter ⟨*P*_2_⟩, which is equal to the second-order coefficient of an ODF of a molecule with cylindrical symmetry, is used as a quantity describing the breadth of ODF.^18^ An example of 3D orientation results is shown in Figure 1e. It should be noted that all vectors have the same length and only vector directions are of significance. Since sections d and e of Figure 1 show results of corresponding sample regions, it is clearly visible how important advantage one gains using the method for 3D orientation determination. Even though this method was developed for FT-IR, the adaptation for O-PTIR and Raman spectra was done by transferring intensities to reach values corresponding to FT-IR absorbance, as it was briefly described in the preprocessing section.

## Supporting Information Available

3D orientation calculation approach; spectroscopic band identification; in-plane orientation (2D) results; 3D orientation results; exemplary polarized FT-IR spectra (PDF)
