# Supplementary material for: Super-Resolved 3D Mapping of Molecular Orientation Using Vibrational Techniques
Source: J Am Chem Soc. 2022 Jul 26;144(31):14278–87. doi: 10.1021/jacs.2c05306 (PMC9376951; doi:10.1021/jacs.2c05306)
Supplement: Supplementary file 1 — ja2c05306_si_001.pdf [file ja2c05306_si_001.pdf]

Supplementary Information: Super-resolved 3D mapping of molecular orientation using vibrational techniques

Paulina Koziol[1,2], Karolina Kosowska[1], Danuta Liberda[1], Ferenc Borondics[3], Tomasz P. Wrobel[1*]

[1] Solaris National Synchrotron Radiation Centre, Jagiellonian University, Czerwone Maki 98, 30-392 Krakow, Poland.
[2] Institute of Physics, Jagiellonian University, Lojasiewicza 11, 30-348 Krakow, Poland
[3] Synchrotron Soleil, L'Orme des Merisiers, Saint-Aubin - BP 48, 91192, Gif-sur-Yvette, France

*email: tomek.wrobel@uj.edu.pl

3D orientation calculations

Following parameters: $\alpha_{1,max}, \eta_{1,max}, \alpha_{1,min}, \eta_{1,min}, \alpha_{2,max}, \eta_{2,max}$ and $\alpha_{2,min}, \eta_{2,min}$, extracted from functions fitted to experimental data as shown in Figure 1, are later used to calculate intermediate parameters, defined as[1]:

$$M \equiv \left(\frac{\alpha_{1,max}}{\alpha_{1,min}} - 1\right) = \frac{3\langle P_2\rangle}{1-\langle P_2\rangle} \sin^2\theta,$$

$$N \equiv \left(\frac{\alpha_{2,max}}{\alpha_{2,min}} - 1\right) = \frac{3\langle P_2\rangle}{1-\langle P_2\rangle} (\cos^2\phi \cos^2\theta + \sin^2\phi),$$

$$\Delta\eta = \eta_{2,max} - \eta_{1,max},$$

$$P = \frac{1}{2}\left[(M+N) + \sqrt{(M-N)^2 + 4MN\cos^2\theta\Delta\eta}\right].$$

One may notice that for $\frac{\alpha_{1max}}{\alpha_{1,min}}$ and $\frac{\alpha_{2,max}}{\alpha_{2,min}}$ ratios in the definition of M and N, $\alpha°$ may be considered as a constant, thus, have no impact in further calculations. Having intermediate parameters and after equations manipulation, it is a straight way to find results for μ_1 and μ_2 orientations along with order parameter[1]:

$$\langle P_2\rangle = \frac{P}{P+3},$$

$$\psi = \eta_{1,max},$$

$$\theta = \sin^{-1}\sqrt{\frac{M}{P}},$$

$$\phi = \cos^{-1}\left[\pm\sqrt{\frac{(P-N)}{M}}\right].$$

The sign in braces of ϕ's formula is defined by the sign of $\tan\Delta\eta$. Analyzing formulas for θ and ϕ, one may notice that ranges of possible results vary within $0 \leq \theta_1 \leq \pi/2$ and $0 \leq \phi_1 \leq \pi$. However, due to the symmetry of transition dipole moments with respect to the polarization plane, there is

another possible solution, defined as $\theta_2 = \pi - \theta_1$ and $\phi_2 = -\phi_1$. Those two solutions are indistinguishable and might be considered as mirror symmetrical with respect to the xy plane. Full derivation of above formulas, along with wider discussion is available in [1]. Nonetheless, all results presented in this publication refer to the first solution with θ_1 and ϕ_1, and one needs to keep in mind that second solution may equally exist.

Spectroscopic bands identification

Table S1. Bands identification for FT-IR.

Wavenumber [cm^{-1}]	Vibration	Comment	κ
1165	ν_s(C-O-C)[2]	-	π/2
1238	ν_{as}(C-O-C)[2]	-	0
1292	ν(CC-O)[2]	Crystalline phase	0
1365	ω(CH_2)[3]	-	0
1720	ν(C=O)[2]	-	π/2

Table S2. Bands identification for O-PTIR.

Wavenumber [cm^{-1}]	Vibration	Comment	κ
1170	ν_s(C-O-C)[2]	-	π/2
1246	ν_{as}(C-O-C)[2]	-	0
1296	ν(CC-O)[2]	Crystalline phase	0
1368	ω(CH_2)[3]	-	0
1736	ν(C=O)[2]	-	π/2

Table S3. Bands identification for Raman.

Raman shift [cm^{-1}]	Vibration	Comment	κ
1037	ν(C-C)[4,5]	-	0
1109	ν(C-C)[4,5]	Crystalline phase	0
1284	ω(CH_2)[4,5]	Crystalline phase	0
1419	δ(CH_2)[4,6]	-	π/2
1724	ν(C=O)[4,6]	-	π/2
2870	ν(CH_2)[7]	-	π/2

In-plane orientation (2D) results

FT-IR and O-PTIR results

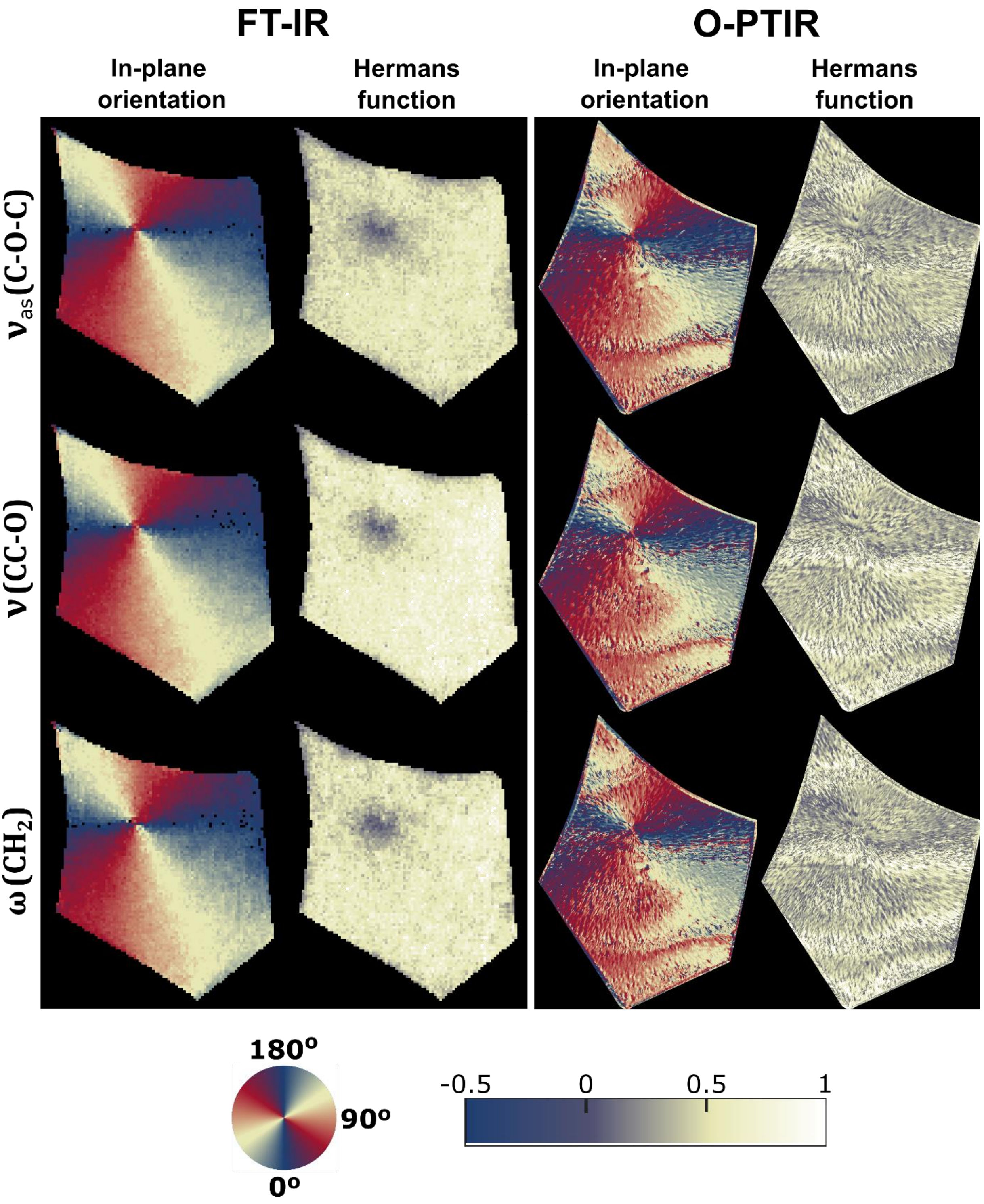

Figure S1. In-plane orientation (2D) and Herman's function results for specific vibration bands, based on FT-IR (left) and O-PTIR (right) methods. Scales presented at the bottom of above figure also apply to figure S2-S3.

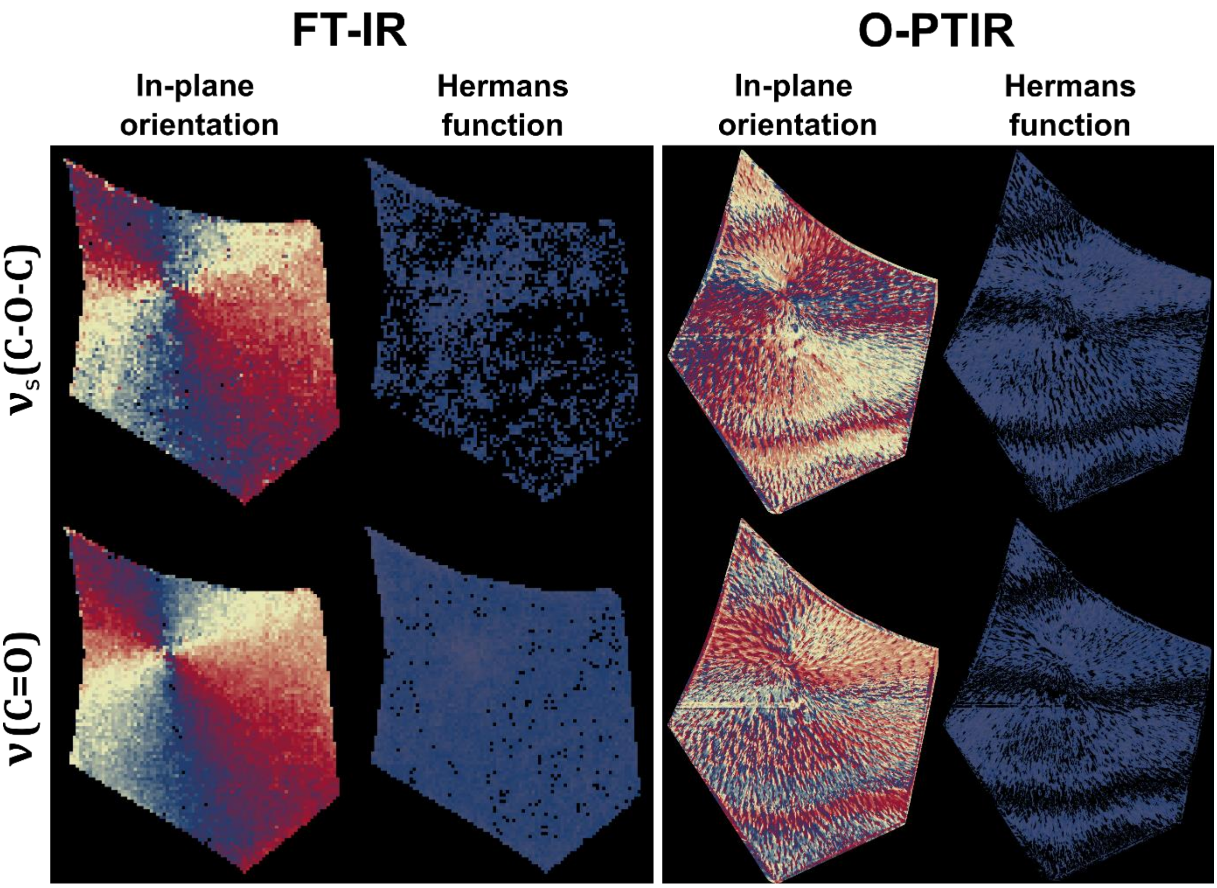

Figure S2. In-plane orientation (2D) and Herman's function results for specific vibration bands, based on FT-IR (left) and O-PTIR (right) methods.

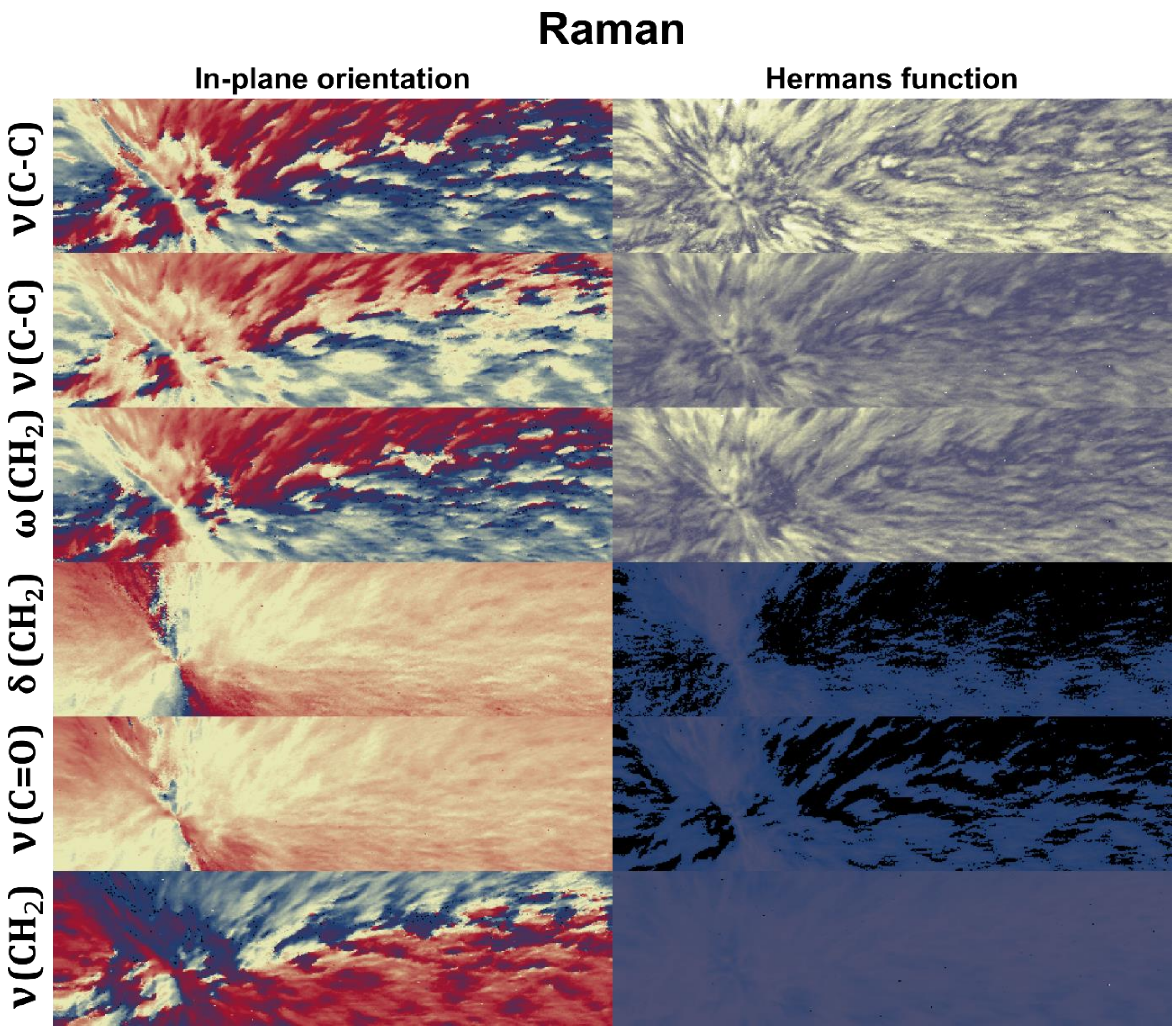

Figure S3. In-plane orientation (2D) and Herman’s function results for specific bands, based on Raman spectroscopy.

3D results

FT-IR

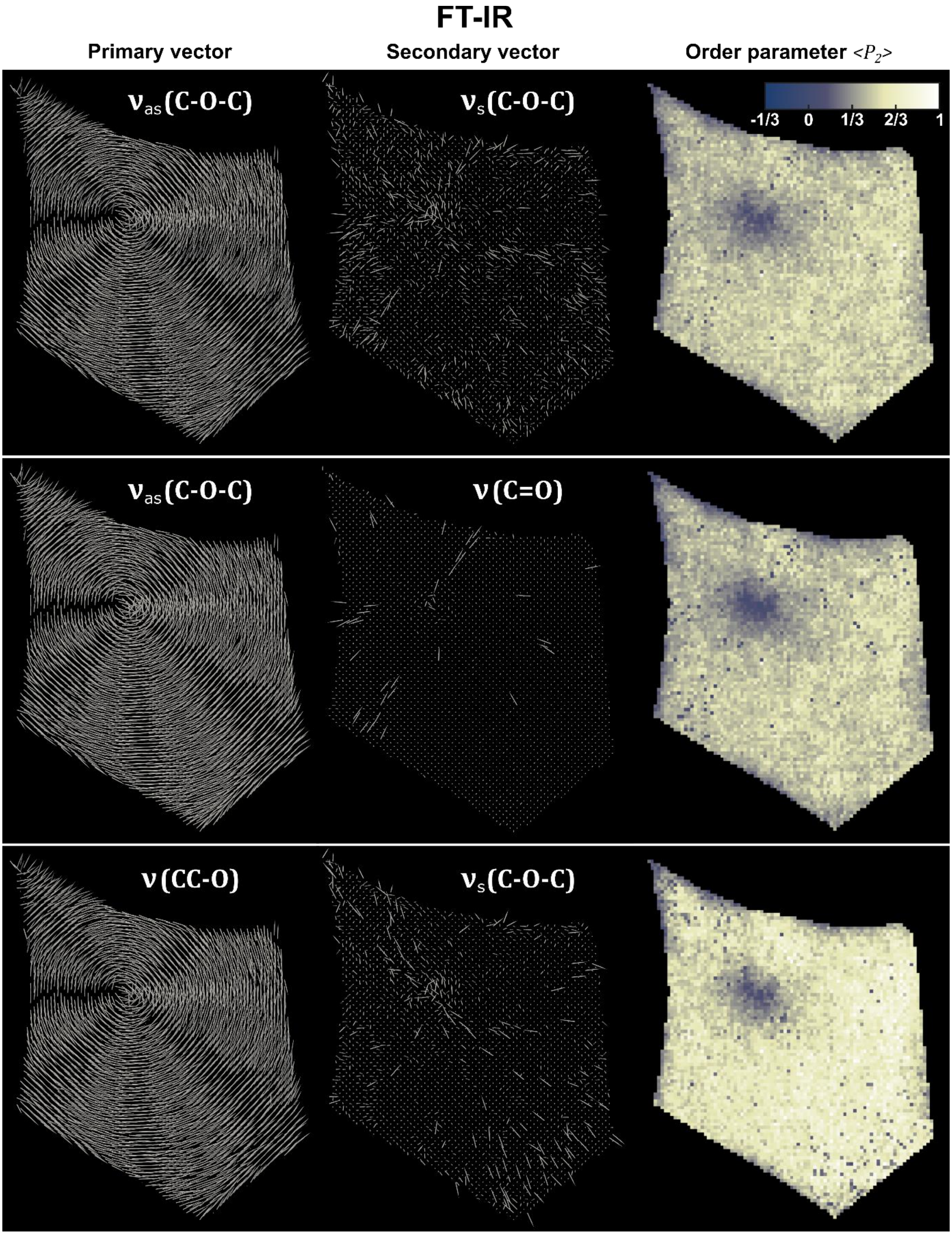

Figure S4. Results of 3D orientation along with order parameter based on FT-IR results. Each row corresponds to analysis done based on pair of perpendicular dipole moments (primary and secondary vectors).

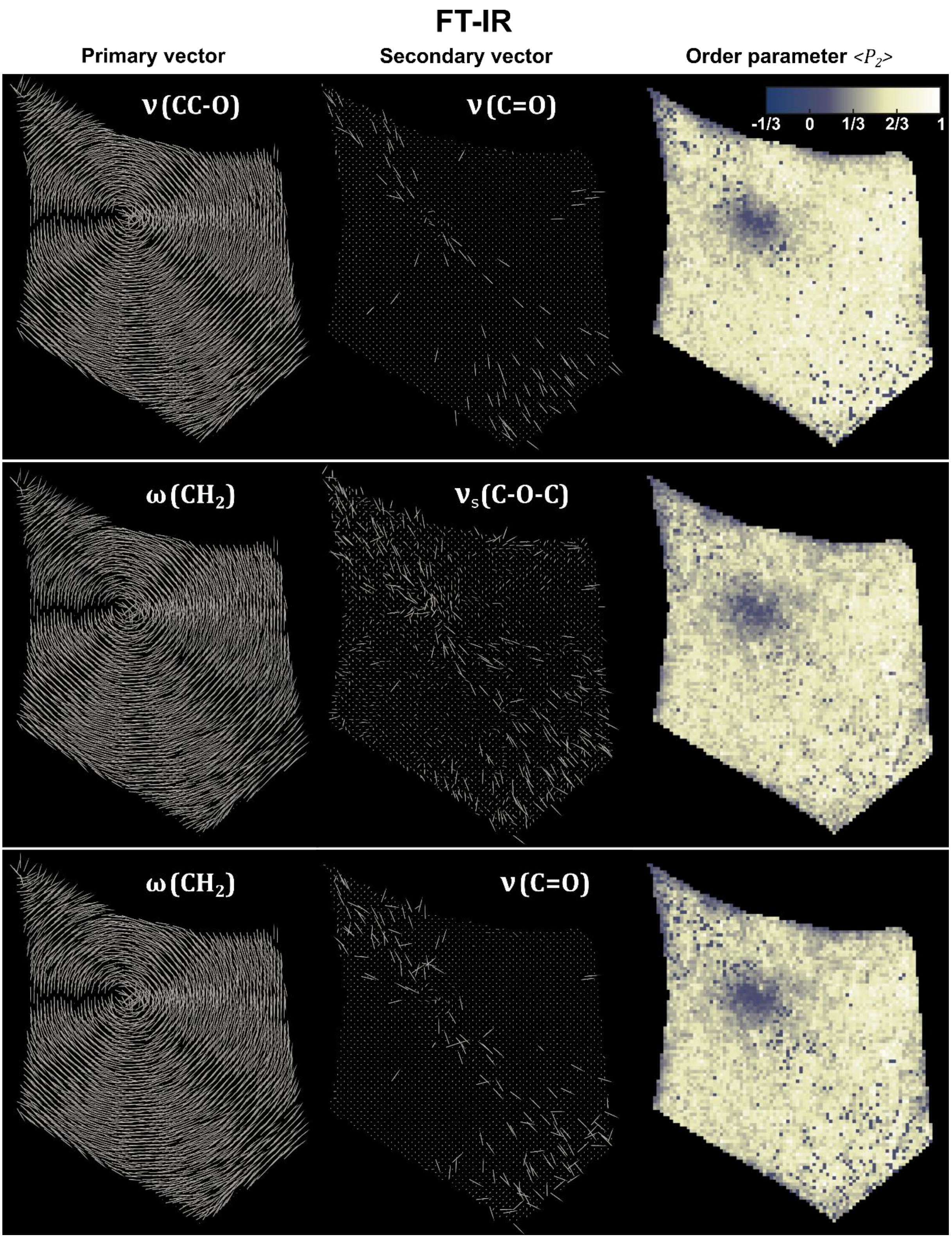

Figure S5. Results of 3D orientation along with order parameter based on FT-IR results. Each row corresponds to analysis done based on pair of perpendicular dipole moments (primary and secondary vectors).

Binned O-PTIR

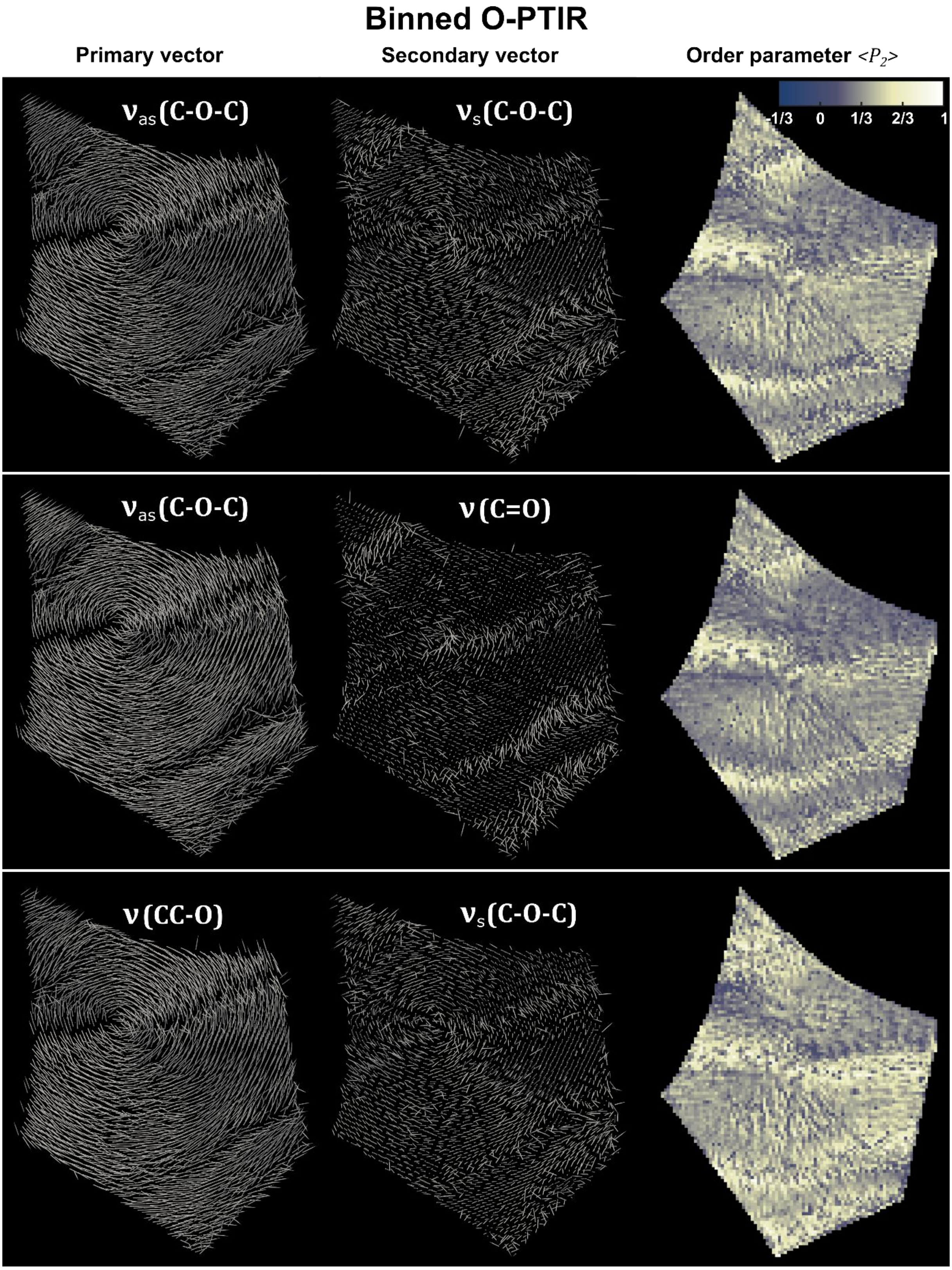

Figure S6. Results of 3D orientation along with order parameter based on O-PTIR results. Each row corresponds to analysis done based on pair of perpendicular dipole moments (primary and secondary vectors). 13x13 pixel region was binned to result in pixel size similar to FT-IR data, allowing straight forward comparison.

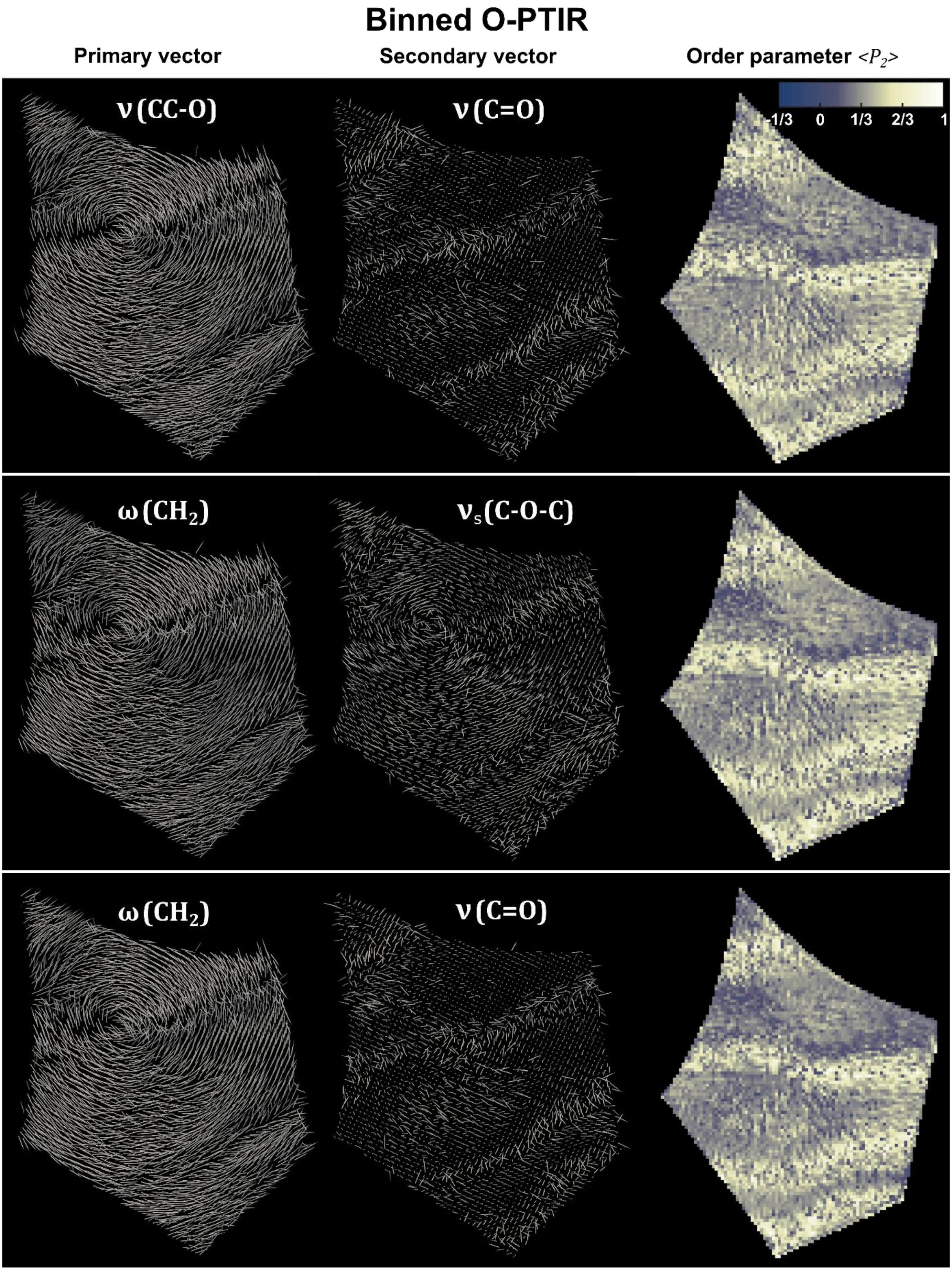

Figure S7. Results of 3D orientation along with order parameter based on O-PTIR results. Each row corresponds to analysis done based on pair of perpendicular dipole moments (primary and secondary vectors). 13x13 pixel region was binned to result in pixel size similar to FT-IR data, allowing straight forward comparison.

Euclidean Distance

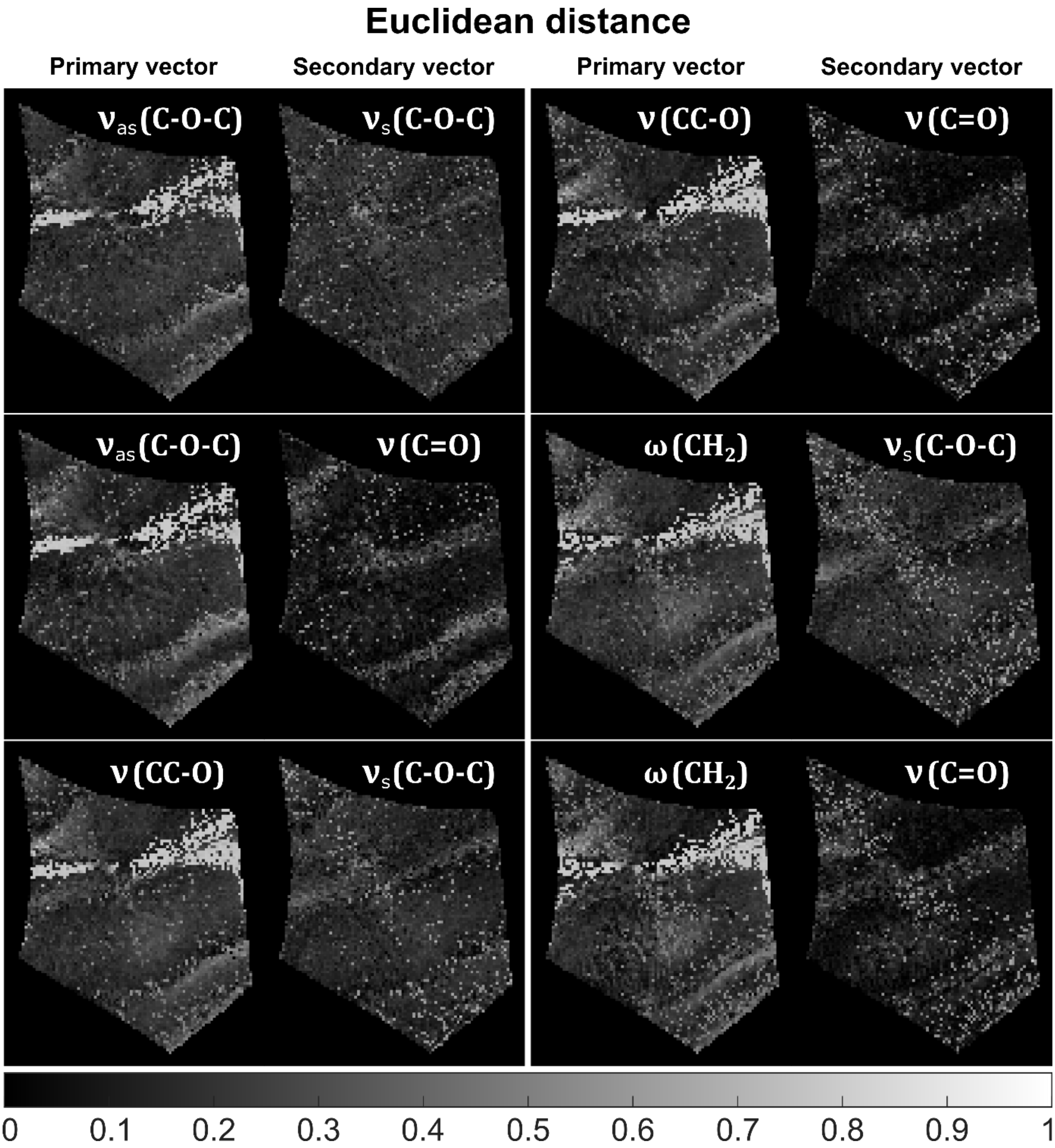

Figure S8. Euclidean distance between corresponding vectors determining 3D orientation results for FT-IR and binned O-PTIR.

O-PTIR

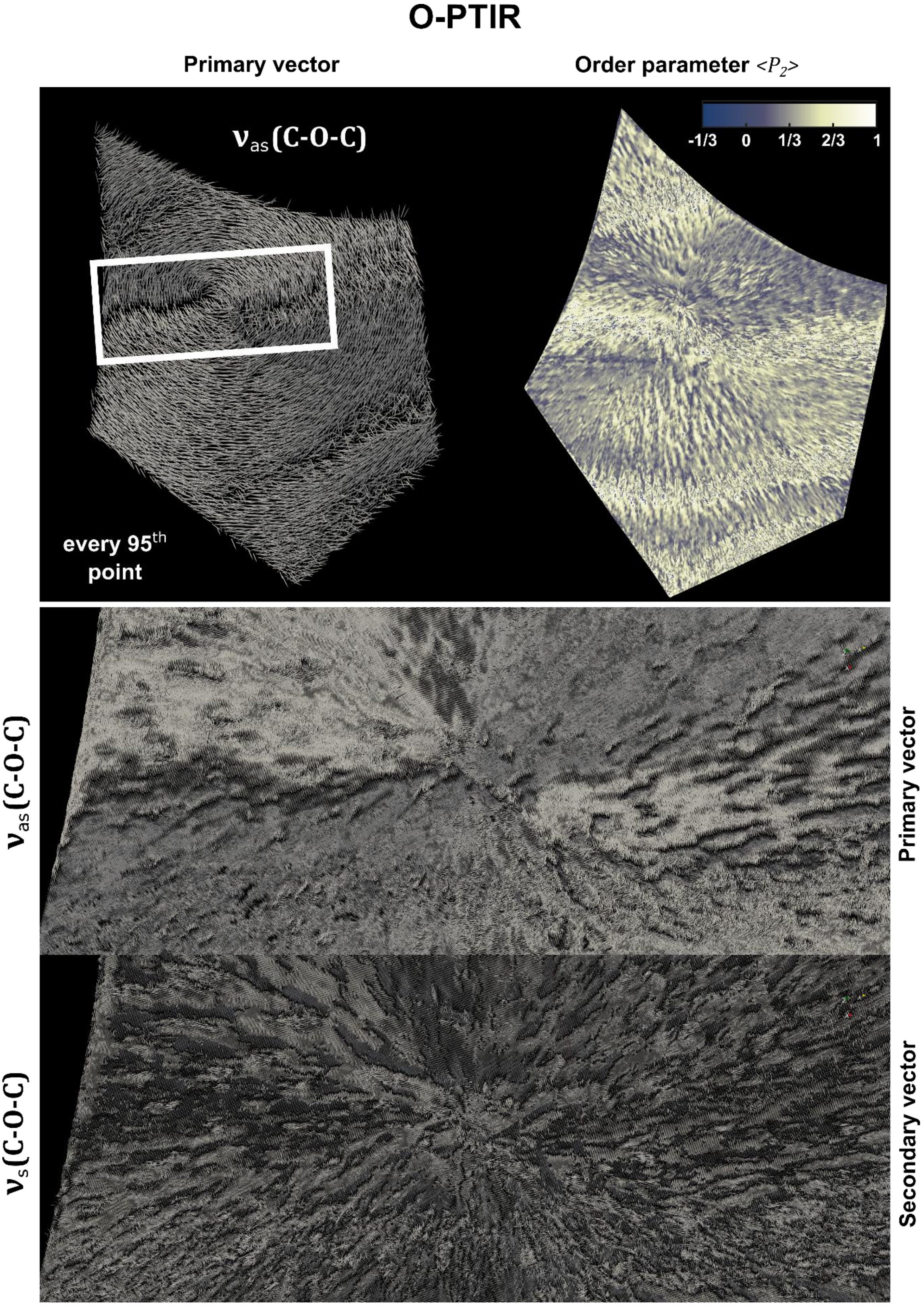

Figure S9. Results of 3D orientation along with order parameter based on O-PTIR. To provide results clarity, primary vector's orientation of only every 95th data point is presented for the full spherulite region (top left part). White box indicates spherulite's region corresponding to orientation results presented in the bottom part.

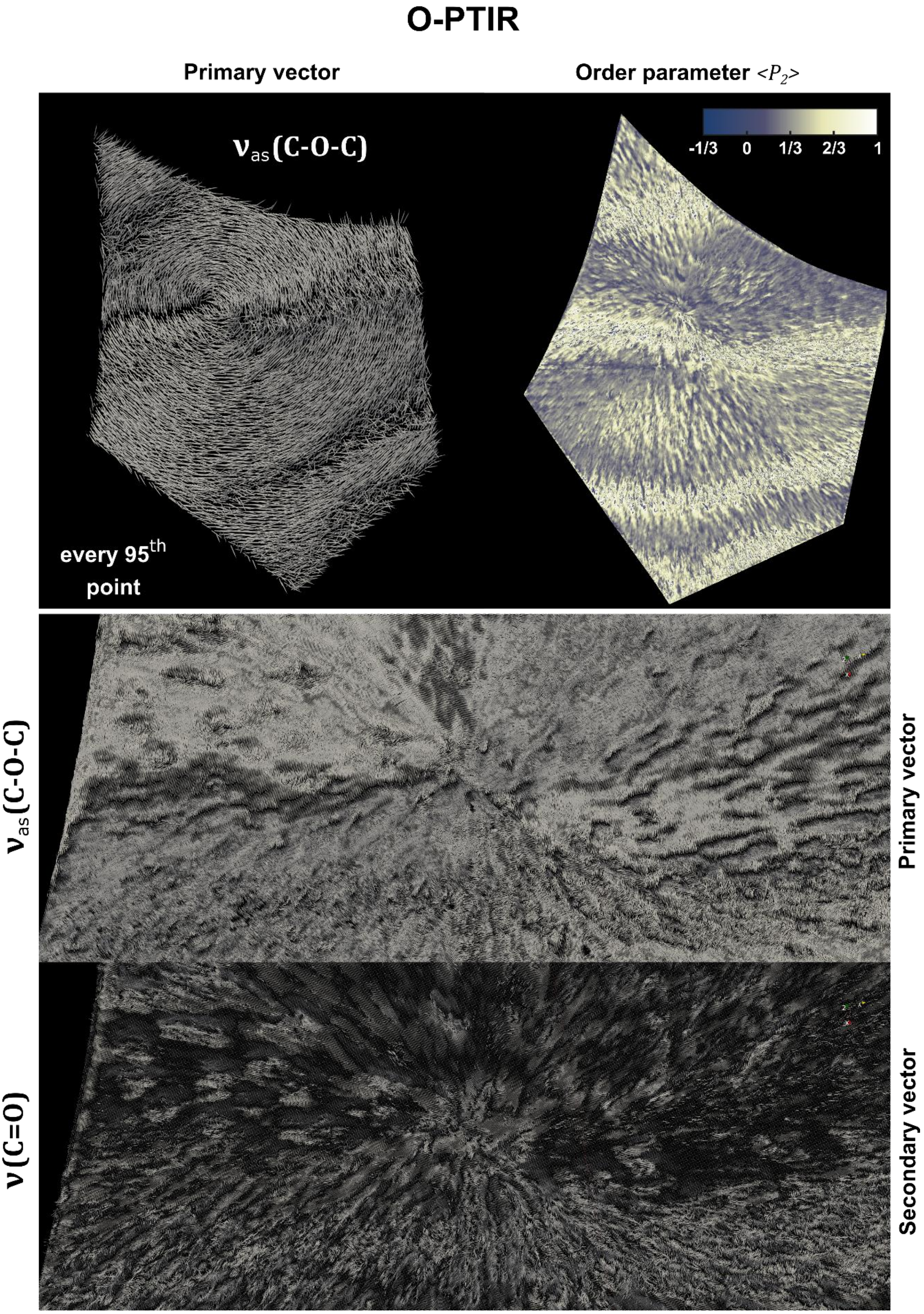

Figure S10. Results of 3D orientation along with order parameter based on O-PTIR. To provide results clarity, primary vector's orientation of only every 95th data point is presented for the full spherulite region (top left part). Results presented in the bottom part of above figure correspond to the spherulite region marked with white box in figure S9.

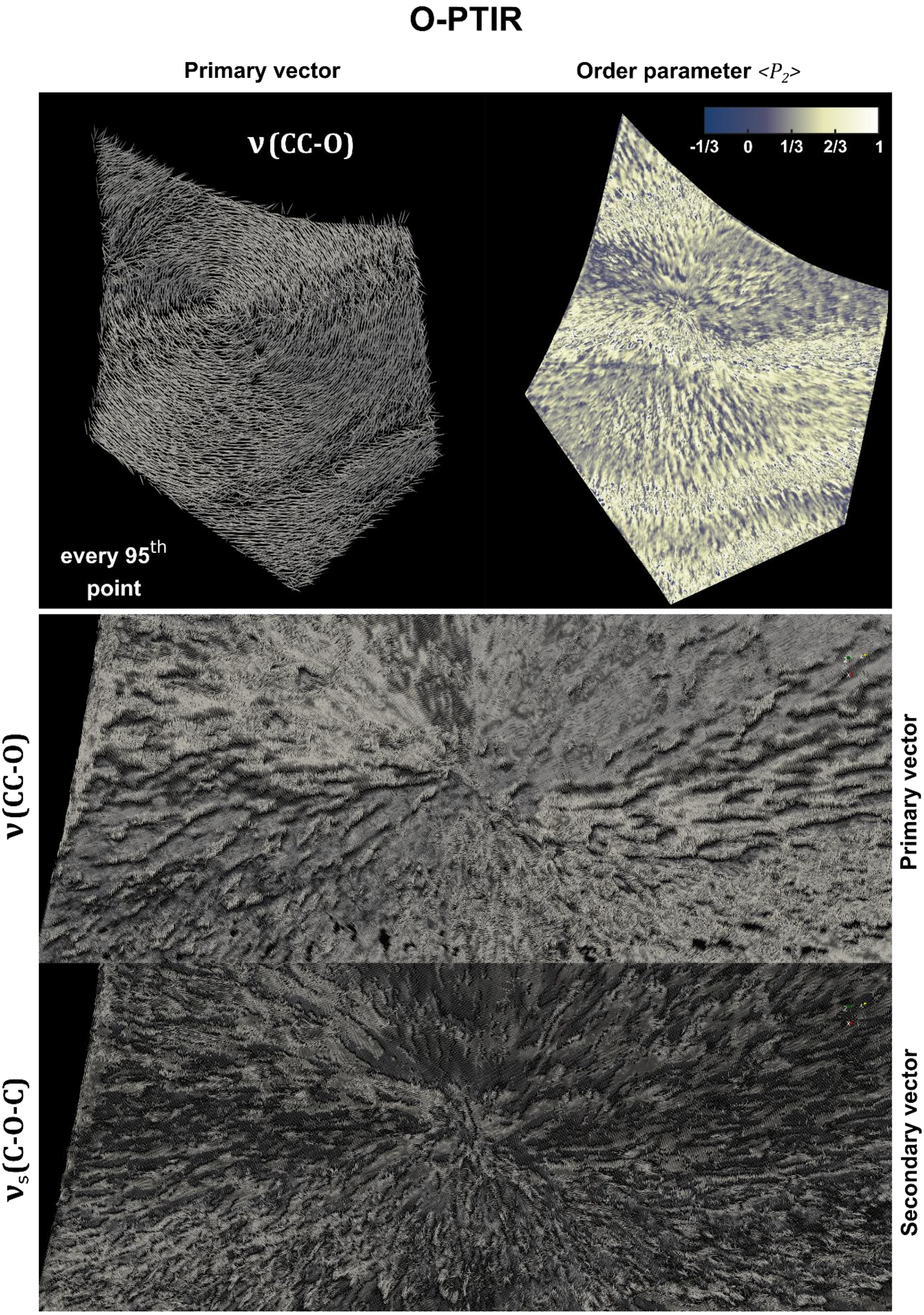

Figure S11. Results of 3D orientation along with order parameter based on O-PTIR. To provide results clarity, primary vector's orientation of only every 95th data point is presented for the full spherulite region (top left part). Results presented in the bottom part of above figure correspond to the spherulite region marked with white box in figure S9.

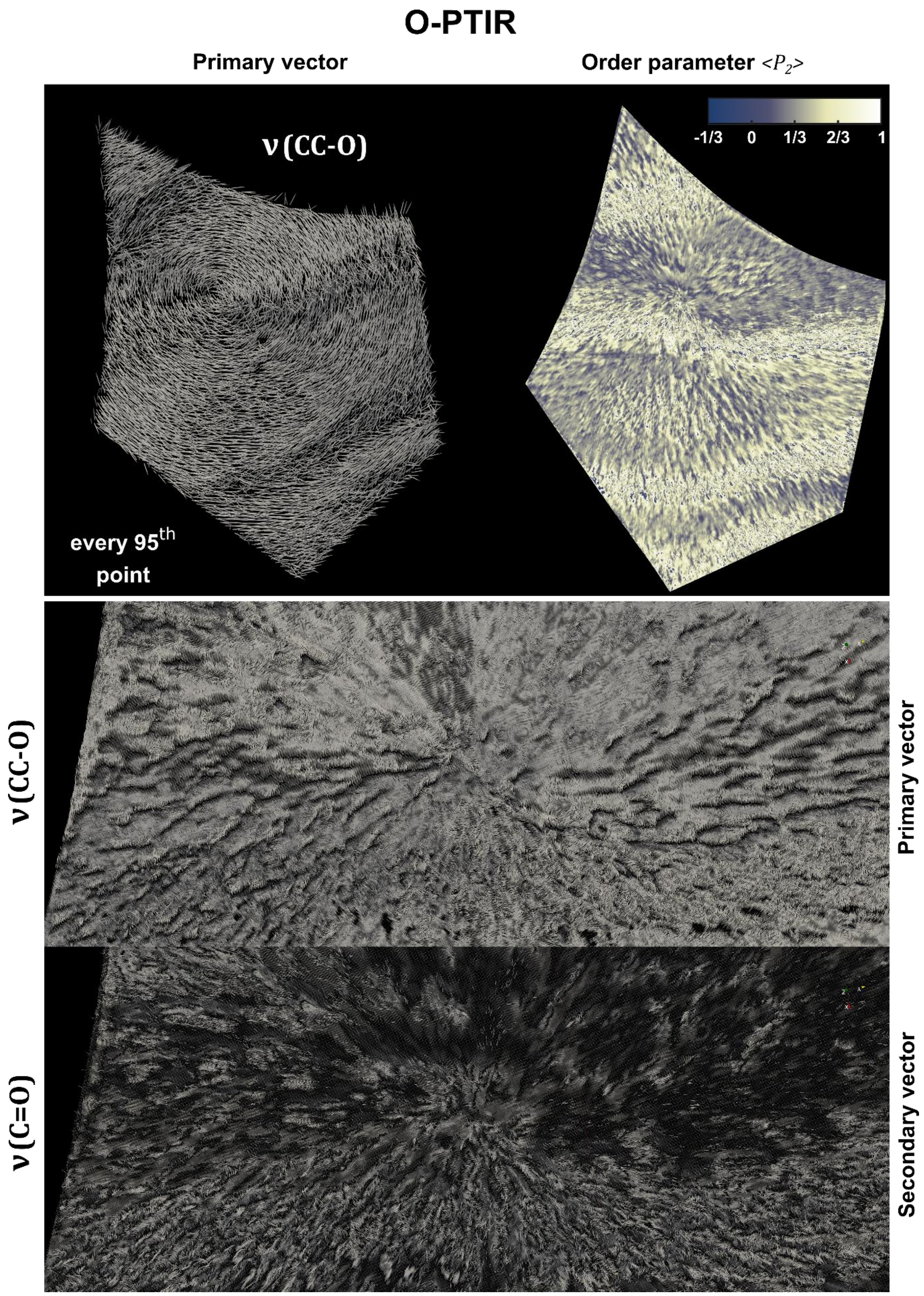

Figure S12. Results of 3D orientation along with order parameter based on O-PTIR. To provide results clarity, primary vector's orientation of only every 95th data point is presented for the full spherulite region (top left part). Results presented in the bottom part of above figure correspond to the spherulite region marked with white box in figure S9.

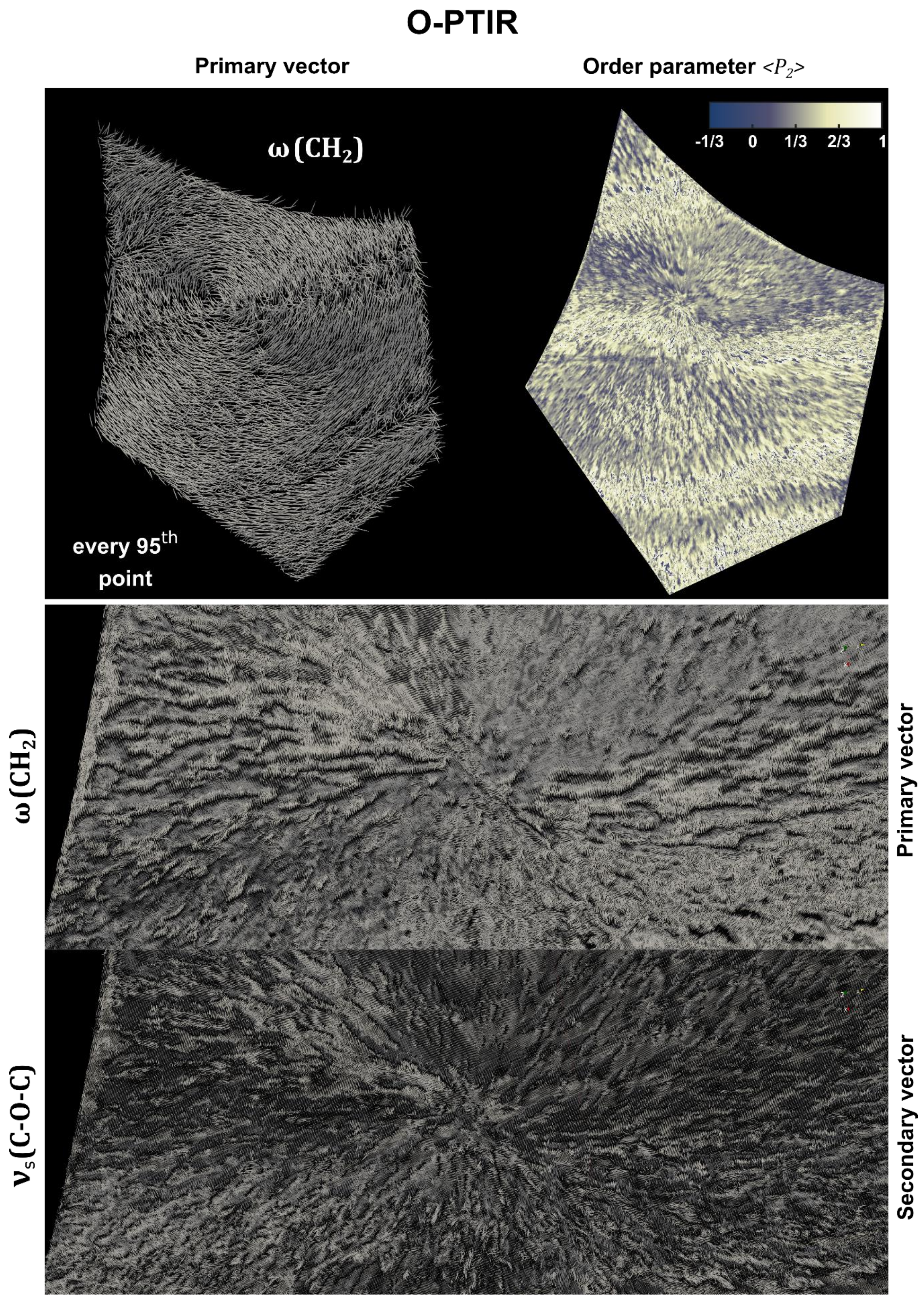

Figure S13. Results of 3D orientation along with order parameter based on O-PTIR. To provide results clarity, primary vector's orientation of only every 95th data point is presented for the full spherulite region (top left part). Results presented in the bottom part of above figure correspond to the spherulite region marked with white box in figure S9.

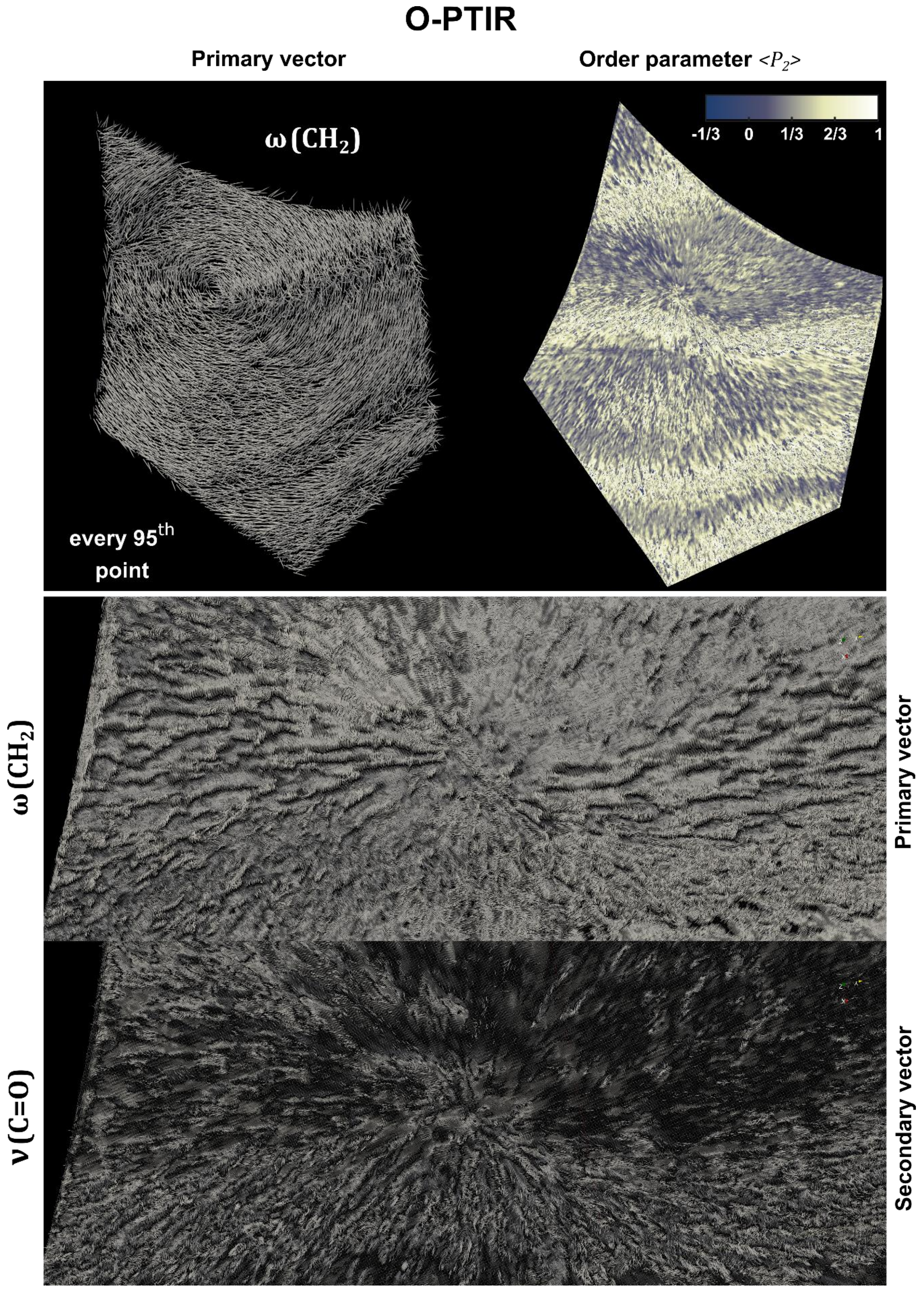

Figure S14. Results of 3D orientation along with order parameter based on O-PTIR. To provide results clarity, primary vector's orientation of only every 95th data point is presented for the full spherulite region (top left part). Results presented in the bottom part of above figure correspond to the spherulite region marked with white box in figure S9.

Raman

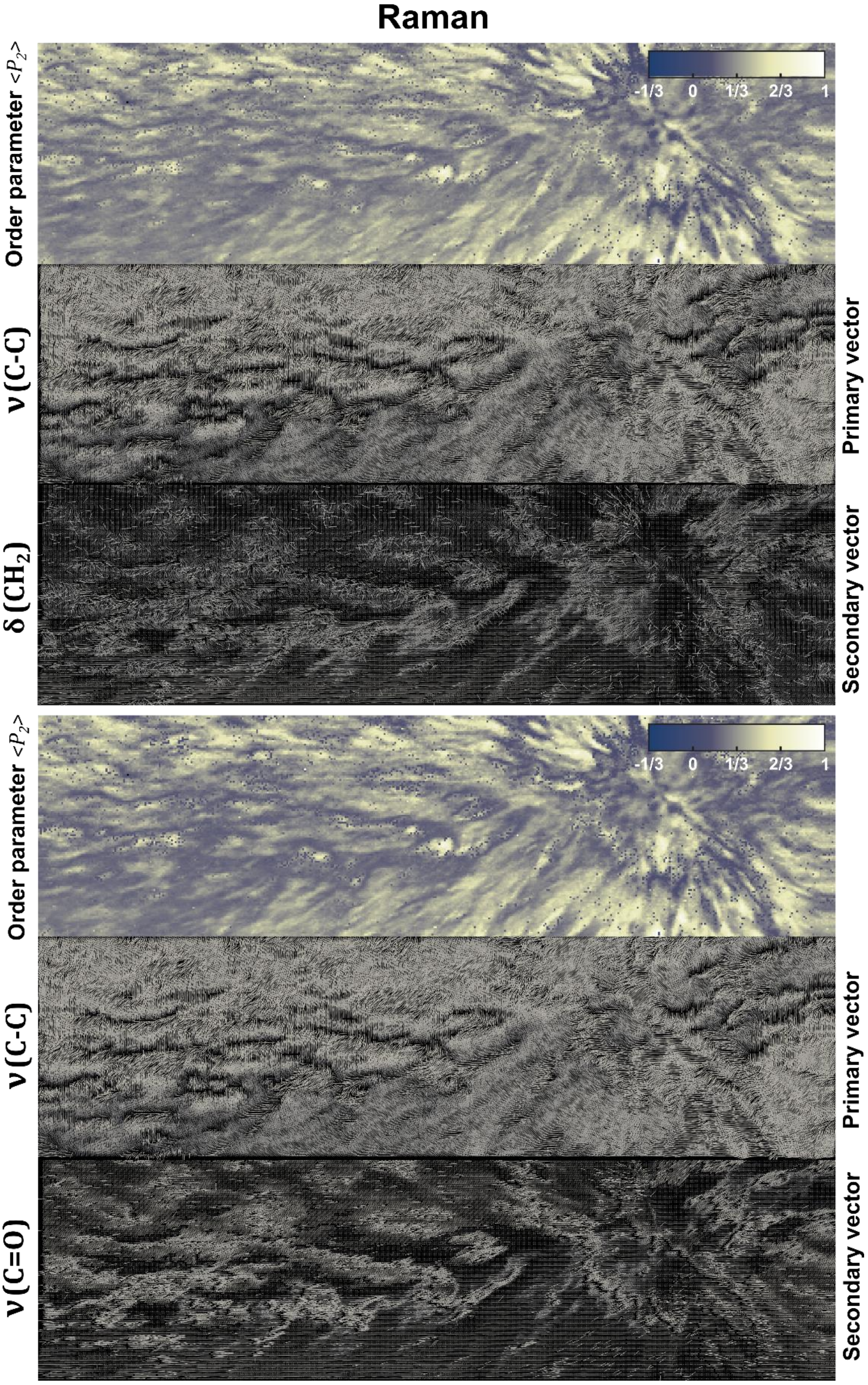

Figure S15. Results of 3D orientation along with order parameter based on Raman results. Each section (top and bottom) corresponds to analysis done based on pair of perpendicular moments (primary and secondary vectors).

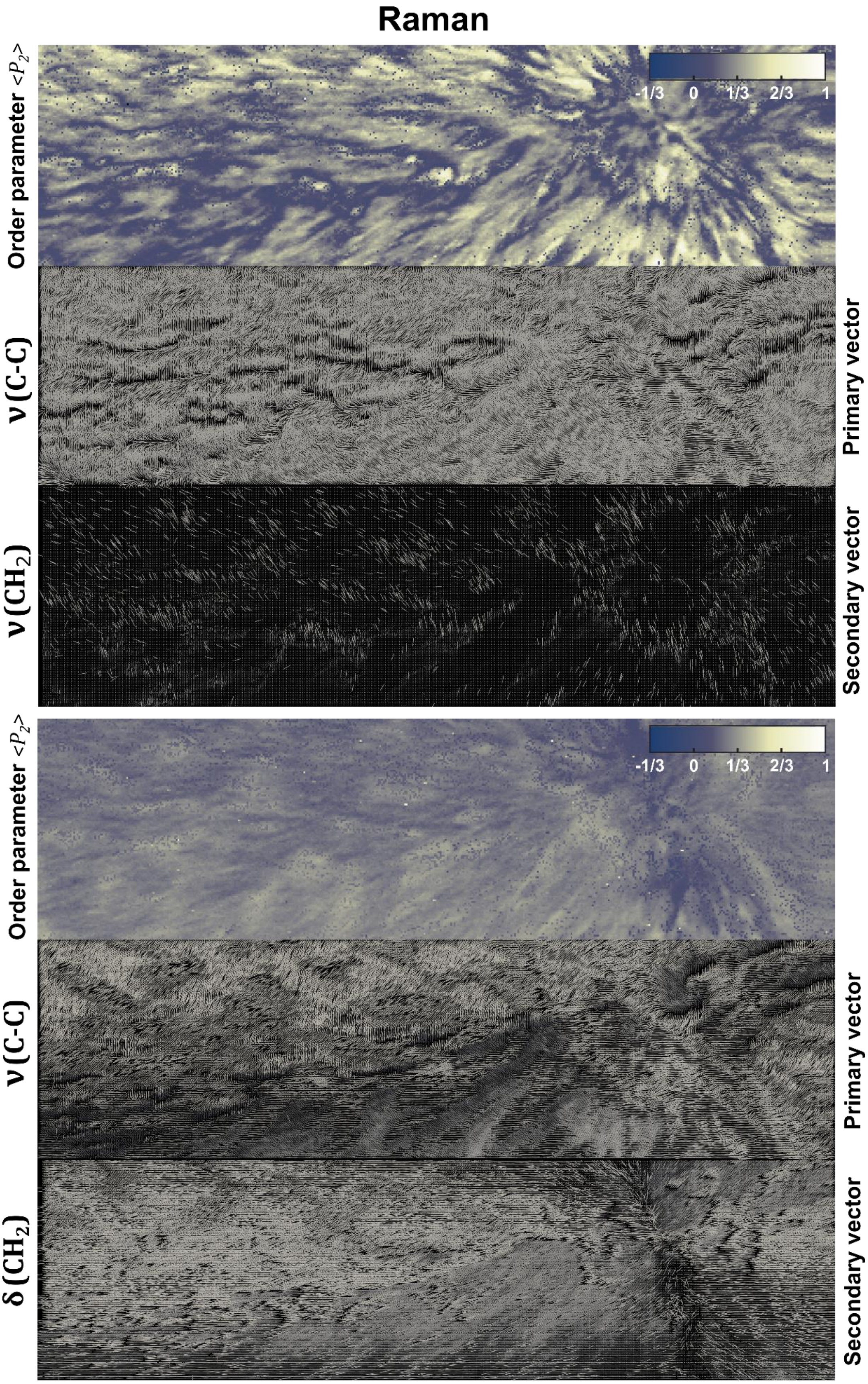

Figure S16. Results of 3D orientation along with order parameter based on Raman results. Each section (top and bottom) corresponds to analysis done based on pair of perpendicular moments (primary and secondary vectors).

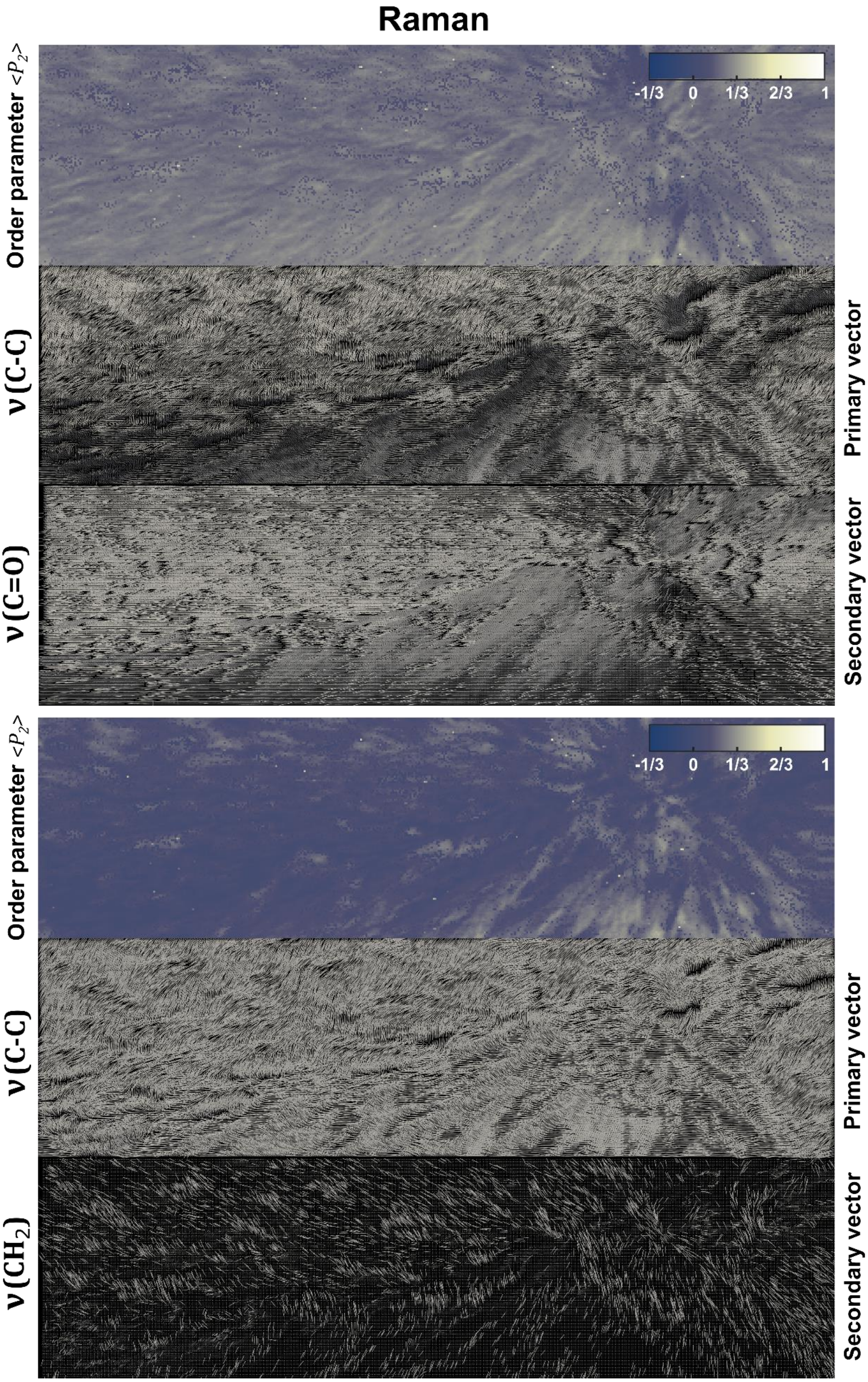

Figure S17. Results of 3D orientation along with order parameter based on Raman results. Each section (top and bottom) corresponds to analysis done based on pair of perpendicular moments (primary and secondary vectors).

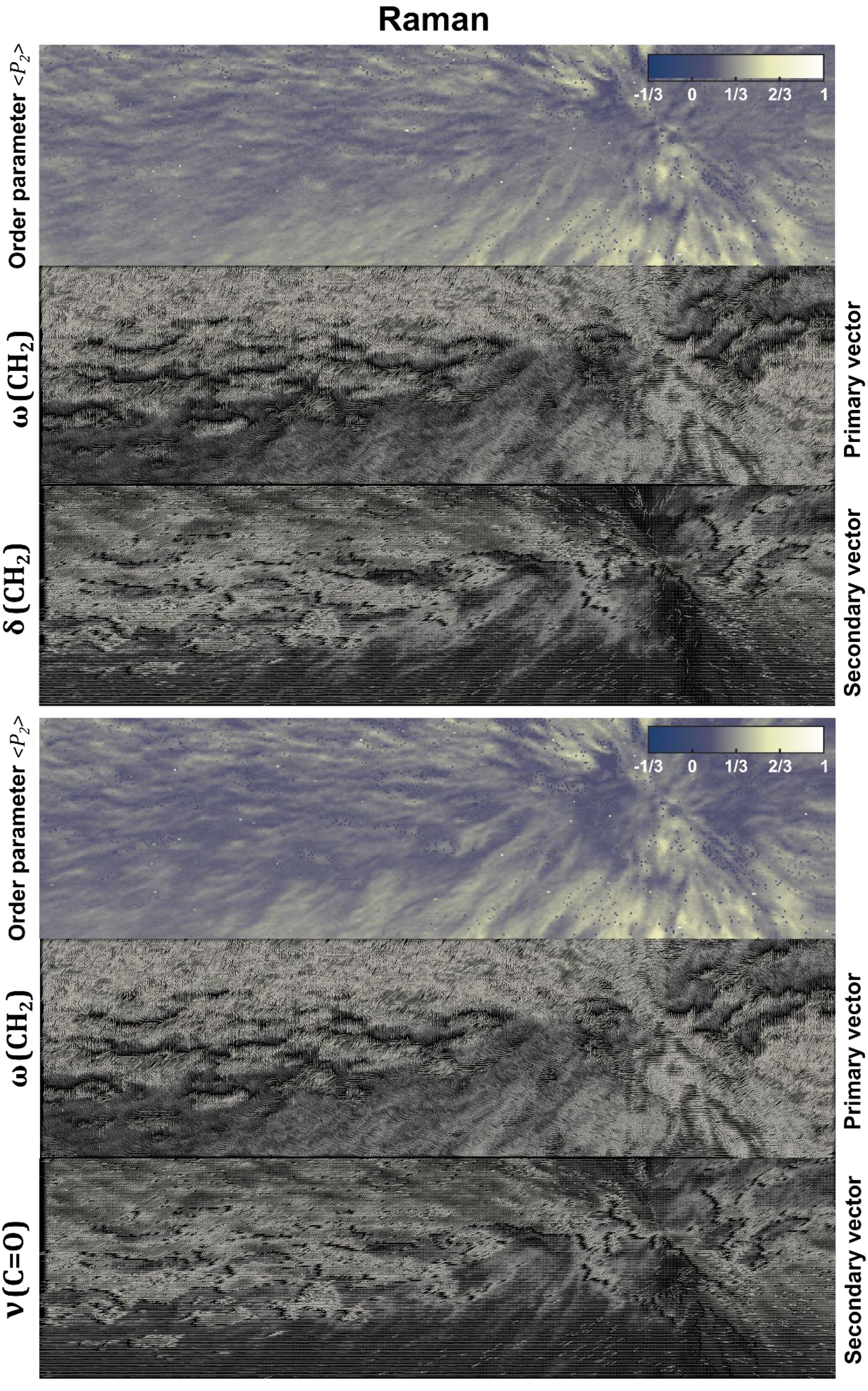

Figure S18. Results of 3D orientation along with order parameter based on Raman results. Each section (top and bottom) corresponds to analysis done based on pair of perpendicular moments (primary and secondary vectors).

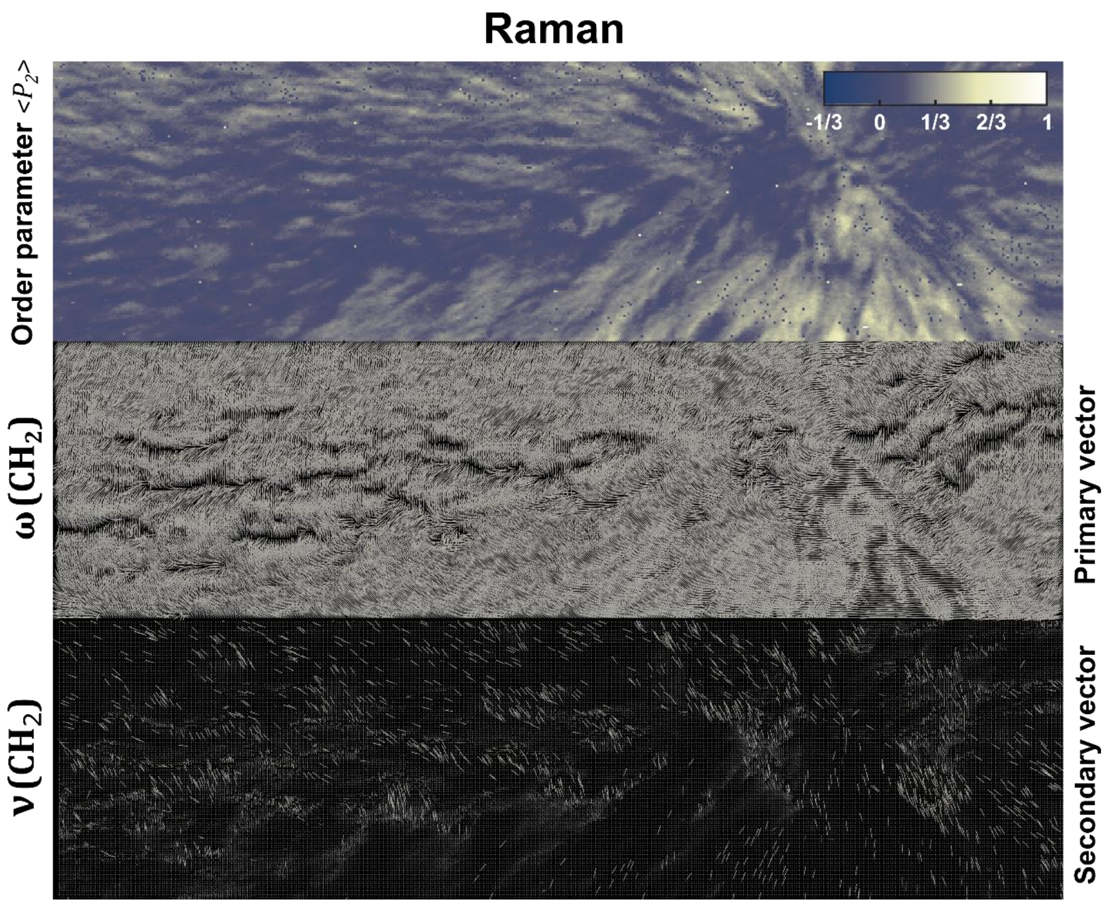

Figure S19. Results of 3D orientation along with order parameter based on Raman results. Analysis was done based on pair of perpendicular moments (primary and secondary vectors).

Spectra

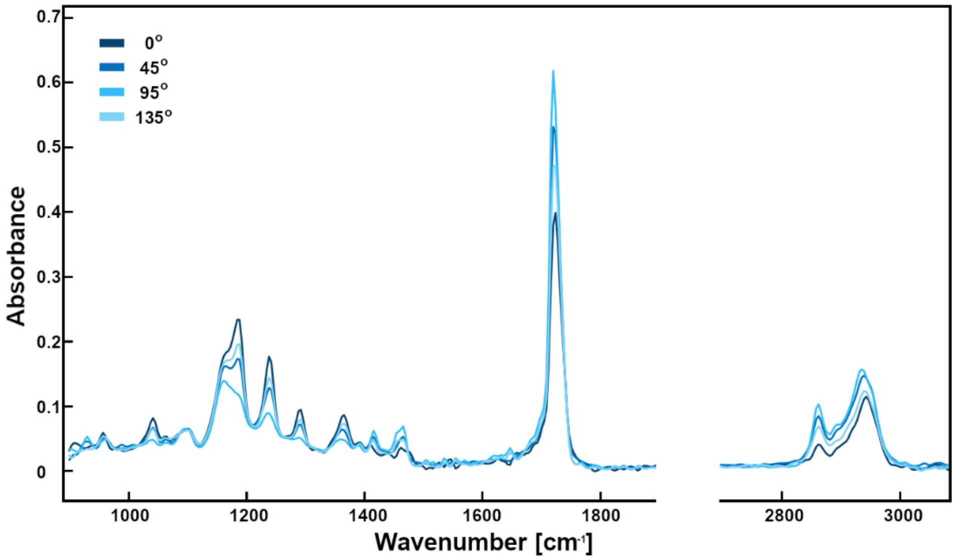

Figure S20. FT-IR measurements of a single pixels with four polarizations.

References

(1) Lee, Y. J. Concurrent Polarization IR Analysis to Determine the 3D Angles and the Order Parameter for Molecular Orientation Imaging. *Optics Express* **2018**, *26* (19), 24577. https://doi.org/10.1364/OE.26.024577.

(2) Elzein, T.; Nasser-Eddine, M.; Delaite, C.; Bistac, S.; Dumas, P. FTIR Study of Polycaprolactone Chain Organization at Interfaces. *Journal of Colloid and Interface Science* **2004**, *273* (2), 381–387. https://doi.org/10.1016/j.jcis.2004.02.001.

(3) Kossack, W.; Kremer, F. Banded Spherulites and Twisting Lamellae in Poly–ε–Caprolactone. *Colloid and Polymer Science* **2019**, *297* (5), 771–779. https://doi.org/10.1007/s00396-019-04503-8.

(4) Kotula, A. P.; Snyder, C. R.; Migler, K. B. Determining Conformational Order and Crystallinity in Polycaprolactone via Raman Spectroscopy. *Polymer (Guildf)* **2017**, *117*, 1–10. https://doi.org/10.1016/j.polymer.2017.04.006.

(5) Smith, G. P. S.; McLaughlin, A. W.; Clarkson, A. N.; Gordon, K. C.; Walker, G. F. Raman Microscopic Imaging of Electrospun Fibers Made from a Polycaprolactone and Polyethylene Oxide Blend. *Vib Spectrosc* **2017**, *92*, 27–34. https://doi.org/10.1016/j.vibspec.2017.05.002.

(6) Kołodziej, A.; Długoń, E.; Świętek, M.; Ziąbka, M.; Dawiec, E.; Gubernat, M.; Michalec, M.; Wesełucha-Birczyńska, A. A Raman Spectroscopic Analysis of Polymer Membranes with Graphene Oxide and Reduced Graphene Oxide. *Journal of Composites Science* **2021**, *5* (1). https://doi.org/10.3390/jcs5010020.

(7) Baranowska-Korczyc, A.; Warowicka, A.; Jasiurkowska-Delaporte, M.; Grześkowiak, B.; Jarek, M.; Maciejewska, B. M.; Jurga-Stopa, J.; Jurga, S. Antimicrobial Electrospun Poly(ε-Caprolactone) Scaffolds for Gingival Fibroblast Growth. *RSC Advances* **2016**, *6* (24), 19647–19656. https://doi.org/10.1039/C6RA02486F.
